# Liquid Biopsy in Gastric Cancer: Analysis of Somatic Cancer Tissue Mutations in Plasma Cell-Free DNA for Predicting Disease State and Patient Survival

**DOI:** 10.14309/ctg.0000000000000403

**Published:** 2021-09-24

**Authors:** Greta Varkalaite, Michael Forster, Andre Franke, Juozas Kupcinskas, Jurgita Skieceviciene

**Affiliations:** 1 Institute for Digestive Research, Lithuanian University of Health Sciences, Kaunas, Lithuania;; 2 Institute of Clinical Molecular Biology, Christian-Albrechts-University of Kiel, Kiel, Germany;; 3 Department of Gastroenterology, Lithuanian University of Health Sciences, Kaunas, Lithuania.

## Abstract

Gastric cancer (GC) diagnosis in late stages and high mortality rates are the main issues that require new noninvasive molecular tools. We aimed to assess somatic mutational profiles in GC tissue and plasma cell-free DNA (cfDNA), evaluate their concordance rate, and analyze the role of multilayer molecular profiling to predict disease state and prognosis.

**Methods:**

Treatment-naive GC patient group (n = 29) was selected. Whole exome sequencing (WES) of GC tissue was performed, and a unique 38-gene panel for deep targeted sequencing of plasma cfDNA was developed. Oncoproteins were measured by enzyme-linked immunosorbent assay, and other variables such as tumor mutational burden and microsatellite instability were evaluated using WES data.

**Results:**

The yield of cfDNA was increased 43.6-fold; the integrity of fragments was decreased in GC compared with controls. WES analysis of cancerous tissue and plasma cfDNA (targeted sequencing) mutational profiles revealed 47.8% concordance. The increased quantity of GC tissue–derived alterations detected in cfDNA was associated with worse patients' survival. Analysis of importance of multilayer variables and receiver operating characteristic curve showed that combination of 2 analytes: (i) quantity of tissue matching alterations and (ii) presence of any somatic alteration in plasma cfDNA resulted in area under curve 0.744 when discriminating patients with or without distant metastasis. Furthermore, cfDNA sequence alterations derived from tumor tissue were detected in patients who had even relatively small GC tumors (T1-T2).

**Discussion:**

Our results indicate that quantitative and qualitative cfDNA mutational profile analysis is a promising tool for evaluating GC disease status or poorer prognosis.

## INTRODUCTION

Gastric cancer (GC) is one of the most common and lethal oncological diseases of the gastrointestinal tract worldwide because it is usually diagnosed at an advanced stage because of asymptomatic course of the disease (1). It is a complex disease arising from the interaction of environmental and host-associated factors (2,3), and conventional diagnostic techniques or current molecular biomarkers have a very limited role for early diagnosis of GC (4,5). Thus, minimally invasive biomarkers that would help to determine specific molecular spectra for diagnostic and prognostic purposes are highly needed.

Improving technologies have enabled a more comprehensive molecular analysis in the body fluids of patients with cancer and have revealed that circulating tumor–derived molecules could provide multilayer molecular information suitable for cancer diagnostics, prognosis, or even response to therapy (6–8). The currently available studies analyzing ctDNA alterations in GC focus on a limited number of well-known oncogenes such as *TP53* (6) and *HER2* (9–11). On the other hand, studies implementing high-throughput technologies such as new generation sequencing (NGS) are still very scarce and have been mostly conducted in Asian populations (12–14).

In this study by using cancer tissue whole exome sequencing (WES), we developed custom 38-gene panel and performed cfDNA deep targeted sequencing in plasma samples. We were able to identify somatic alterations in cfDNA in a solid proportion of the patients with GC, including patients with early disease stages. Moreover, we performed multicomponent analysis for GC using machine learning on various analytes including cfDNA and oncoproteins. Our study suggests that qualitative and quantitative analysis of somatic variants in the plasma cfDNA might be a promising approach when discriminating patients based on disease state and even predict survival.

## MATERIALS AND METHODS

### Patient samples

**Figure 1.**
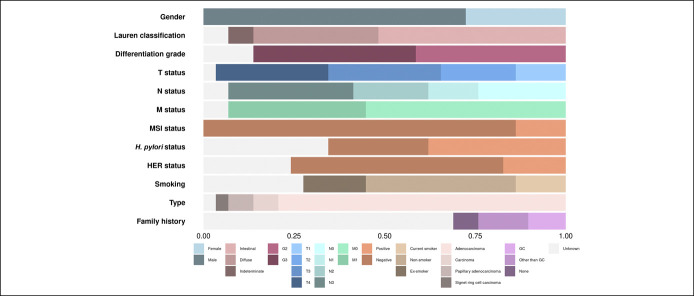
Characteristics of patients with GC (n = 29). A bar graph representing the proportion of patients in each section. GC, gastric cancer; HER, human epidermal growth factor receptor; MSI, microsatellite instability.

Treatment-naive GC patients (n = 29) were recruited at the Department of Gastroenterology, Lithuanian University of Health Sciences Hospital during the period of 2015–2018. Clinical and demographic characteristics of patients are summarized in Figure 1 (see also Supplementary Table 1, Supplemental Digital Content 1, http://links.lww.com/CTG/A679). Paired tissue and plasma samples were collected at the same time point. Tumor tissue samples were obtained from the primary lesion during gastroscopy or surgical tumor removal. Peripheral blood was collected using K_2_EDTA tubes (10 mL; Becton, Dickinson and Company, Franklin Lakes, NJ) for cfDNA extraction (double centrifugation protocol within 2 hours of blood draw) and serum separator tubes (5 mL; Becton, Dickinson, and Company) for serum separation. The control group (n = 20) consisted of self-reported healthy subjects without a history of cancer. All subjects provided written informed consent. Research was approved by the Kaunas Regional Biomedical Research Ethics Committee (No. BE-2-10, May 8, 2011, and No. BE-2-31, June 5, 2018, Kaunas, Lithuania).

### Isolation of nucleic acids

Genomic DNA (gDNA) from the primary GC lesion was isolated using the AllPrep DNA/RNA Mini Kit (Qiagen, Hilden, Germany), and gDNA from white blood cells (WBC) was isolated using salting-out method. Total circulating nucleic acids from plasma were extracted using QIAamp Circulating Nucleic Acid isolation kit (Qiagen). All isolations of nucleic acids were performed according to the manufacturers' protocols. cfDNA yield and fragment size were evaluated using TapeStation 2200 system (Agilent Technologies, Santa Clara, CA). Tumor cfDNA fraction was calculated according to mean mutant allele frequency (MAF) in each patient's plasma sample.

### Library preparation of whole exome and targeted NGS

Pair-end (2× 100 bp) sequencing libraries of GC tissue and matched WBC samples were constructed using the Illumina TruSeq Nano DNA Library Prep Kit (Illumina, San Diego, CA) according to the manufacturer's recommendations. gDNA libraries for exome sequencing were captured using the Integrated DNA Technologies xGen Exome Research Panel and hybridization reagents (Integrated DNA Technologies, Coralville, IA). Pair-end (2× 150 bp) sequencing libraries from plasma cfDNA samples were constructed using TruSight Oncology Unique Molecular Identifier (UMI) Reagents (Illumina). cfDNA was captured using Integrated DNA Technologies xGen Custom Panel consisting of 38 GC-associated mutated genes (see Supplementary Table 2, Supplemental Digital Content 2, http://links.lww.com/CTG/A680). All libraries were sequenced on the NovaSeq 6000 platform (Illumina) according to the manufacturer's instructions. The on-target sequence depth metrics are presented in Supplemental Table 3 (see Supplementary Digital Content 3, http://links.lww.com/CTG/A681).

### Variant calling and development of custom gene panel for targeted sequencing

The GATK Best Practices paired-sample workflow (15) for somatic short variant discovery was used for the GC tissue exome analysis (human genome reference build hg19). Variants were called using GATK4 Mutect2 and annotated using Ensembl-VeP (v96.0) (16). Microsatellite instability (MSI) from WES data was evaluated using MSIsensor (17). Tumor mutational burden (TMB) was defined as the quantity of somatic mutations in the coding region per megabase (Mb) (18).

Filtering of somatic variants and selection of GC-related genes for cfDNA custom targeted sequencing panel was performed using following criteria: (i) prevalence of the mutation in general population <1%; (ii) protein coding nonsynonymous, annotated as having high impact; (iii) Combined Annotation Dependent Depletion score >30; (iv) excluding variants that are present in 100% of the samples; and (v) variant supported with coverage ≥2 in both forward and reverse directions.

Plasma cfDNA targeted sequencing analysis was performed using the Illumina UMI Error Correction App (v1.0.0.1), and variants were called using GATK4 Mutect2 and annotated using Ensembl-VeP (v96.0) or SnpEff (v4.3.1t) (19). All detected somatic variants were validated using the integrative genomics viewer (v2.5.3) (20).

### Assessment of serum Carcinoembryonic Antigen, CA 19-9, and Cancer Antigen 72-4 level

Serum level of oncoproteins was measured by enzyme-linked immunosorbent assay (ELISA): Human Carcinoembryonic Antigen (CEA) ELISA Kit (ab99992; Abcam, Cambridge, UK), Human Cancer Antigen CA 19-9 ELISA Kit (ab108642; Abcam), and Human Cancer Antigen 72-4 (Tumor Marker CA724) ELISA Kit (E-EL-H0613; Elabscience, Wuhan, China). All analytical procedures were performed according to manufacturers' instructions.

### Statistical analysis

Statistical analysis and data visualization was performed using R Studio (R version 3.3.3). Comparison of total cfDNA yield was evaluated by 2-sided *t* test or Mann-Whitney *U* test depending on the data distribution. Correlation analysis was performed using the Spearman rank-order correlation analysis. Multivariate comparison was performed using ANOVA, and 2 groups were compared using χ^2^ or Fisher exact tests (2-sided). MAF analysis was conducted using maftools package (Bioconductor) (21). Gene list pathway enrichment analysis was performed using the PANTHER Gene List Analysis tool (22). Random forest analysis of the prediction variables' importance was performed using the Boruta and randomForest packages (23,24). Survival analysis was performed using the Kaplan-Meier method and Cox proportional hazards model.

## RESULTS

### Total cfDNA yield and size of the fragments differ between GC cases and controls

**Figure 2.**
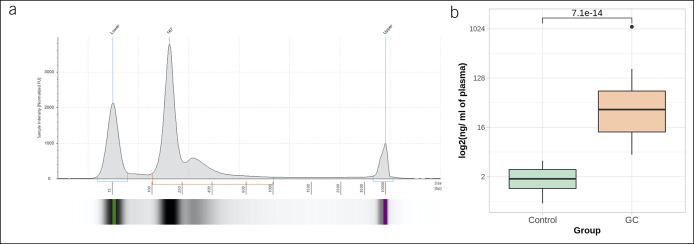
(**a**) Representative electropherogram of cfDNA sample. Fragment size of cfDNA ranges from 100 to 1,000 bp, with the main peak around 170 bp; (**b**) yield of plasma total cfDNA (ng per ml of blood plasma) in control (n = 20) and GC (n = 29) samples. A 43.6-fold increase of cfDNA yield was determined in the GC patient group (*P* = 7.07 × 10^−14^). Results are shown on logarithmic scale. cfDNA, cell-free DNA; GC, gastric cancer.

Total cfDNA yield (fragments from 100 to 1,000 bp, Figure 2a) was compared with GC clinical features and patients' characteristics. As expected, a significantly higher amount of total cfDNA was detected in patients with GC (87.59 ng per ml of plasma) compared with controls (2.01 ng per ml of plasma) (W = 0, *P* = 7.07 × 10^14^) (Figure 2b). Moreover, the analysis of total cfDNA yield revealed positive significant correlation with serum CEA levels (see Supplementary Figure 1, Supplemental Digital Content 4, http://links.lww.com/CTG/A682).

**Figure 3.**
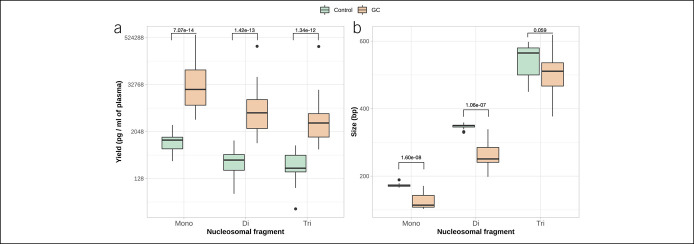
(**a**) Yield of mononucleosomal, dinucleosomal, and trinucleosomal fragments (pg per ml of plasma) in control and GC patients' groups. Statistically significant increase of all cfDNA peaks was observed for patients with GC (*P* = 7.07 × 10^−14^, *P* = 1.42 × 10^−13^, and *P* = 1.34 × 10^−12^, mononucleosomal, dinucleosomal, and trinucleosomal peaks, respectively); (**b**) size of mononucleosomal, dinucleosomal, and trinucleosomal fragments of cfDNA in control and GC patients' groups. Mononucleosomal and dinucleosomal cfDNA peaks of the patients with GC were significantly shorter compared with control cfDNA samples (*P* = 1.60 × 10^−8^ and *P* = 1.06 × 10^−7^, respectively). cfDNA, cell-free DNA; GC, gastric cancer.

Analysis of total cfDNA fragmentation revealed that the yield of all nucleosomal fragments was increased in the GC group compared with control: (i) mononucleosomal fragments: 61,572.60 vs 1,193.94 pg/mL (W = 0, *P* = 7.07 × 10^−14^); (ii) dinucleosomal fragments: 21,373.82 vs 437.80 pg/mL (W = 1, *P* = 1.42 × 10^−13^); and (iii) trinucleosomal fragments: 16,086.52 vs 367.03 pg/mL (W = 5, *P* = 1.34 × 10^−12^) (Figure 3a). The length of the fragments was shorter in the GC group compared with control: (i) mononucleosomal: 73 vs 125 bp (W = 568, *P* = 1.60 × 10^−8^) and (ii) dinucleosomal: 349 vs 259 bp (W = 432.5, *P* = 1.06 × 10^−7^) (Figure 3b).

### Custom gene panel developed according to the mutational spectra of GC tissue

**Figure 4.**
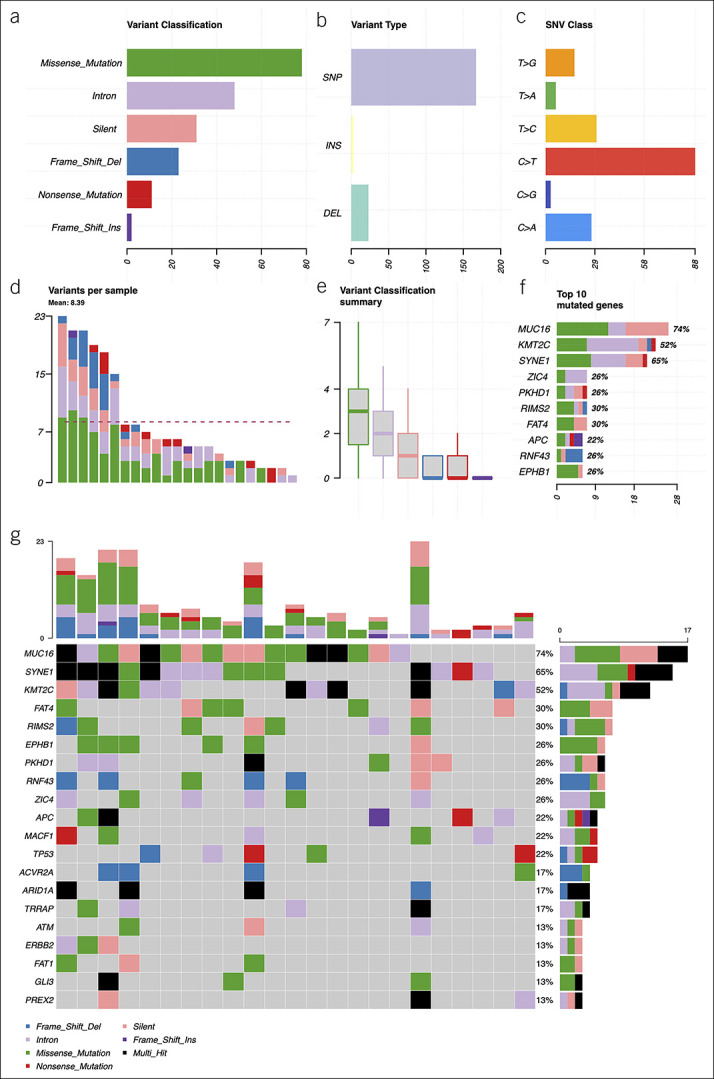
Summary of the mutational spectra (only genes from custom gene panel) in gastric cancer tissue samples: (**a**) absolute variant class values, the most common variant class was missense mutations; (**b**) absolute variant type values, the most common variant type detected was SNPs; (**c**) distribution of various SNV substitutions, C > T substitutions were detected the most frequently; (**d**) absolute numbers of variants per sample, the dashed line shows the mean quantity of somatic variants per sample (8.39); (**e**) mean distribution of variant classes per sample, on average, missense mutations were most frequent; (**f**) top 10 mutated genes, x axis: absolute numbers (samples), percentages calculated from all somatic variants detected; and (**g**) oncoplot of the mutated genes in gastric cancer tissue samples, showing mutated genes and distribution of variant classes per sample. Color codes in (**d-g**) graphs are the same as in (**a**). DEL, deletion; INS, insertion; SNP, single nucleotide polymorphism; SNV, single nucleotide variant.

The GC tissue mutational spectra from WES data are presented in Supplementary Figure 2 (see Supplemental Digital Content 5, http://links.lww.com/CTG/A683). In total, 23 of 29 patients with GC (79.31%) had somatic mutations that passed the previously described selection criteria. On average, alteration-positive tissue samples had 8.4 somatic mutations (range from 1 to 23) in genes included in our custom panel. Variant allele frequencies (VAFs) ranged from 2.8% to 87.1%. Distribution of variant classifications and types is presented in Figure 4a,b. The top 10 most frequently mutated genes are shown in Figure 4f. Based on WES results, a 38-gene panel for very deep targeted sequencing of plasma cfDNA was designed. All somatic mutations of 38 genes included in our panel in tissue samples are presented in Supplementary Table 4 (see Supplemental Digital Content 6, http://links.lww.com/CTG/A684).

### Mutational spectra of plasma cfDNA are associated with tumor size and survival of the patients with GC

**Figure 5.**
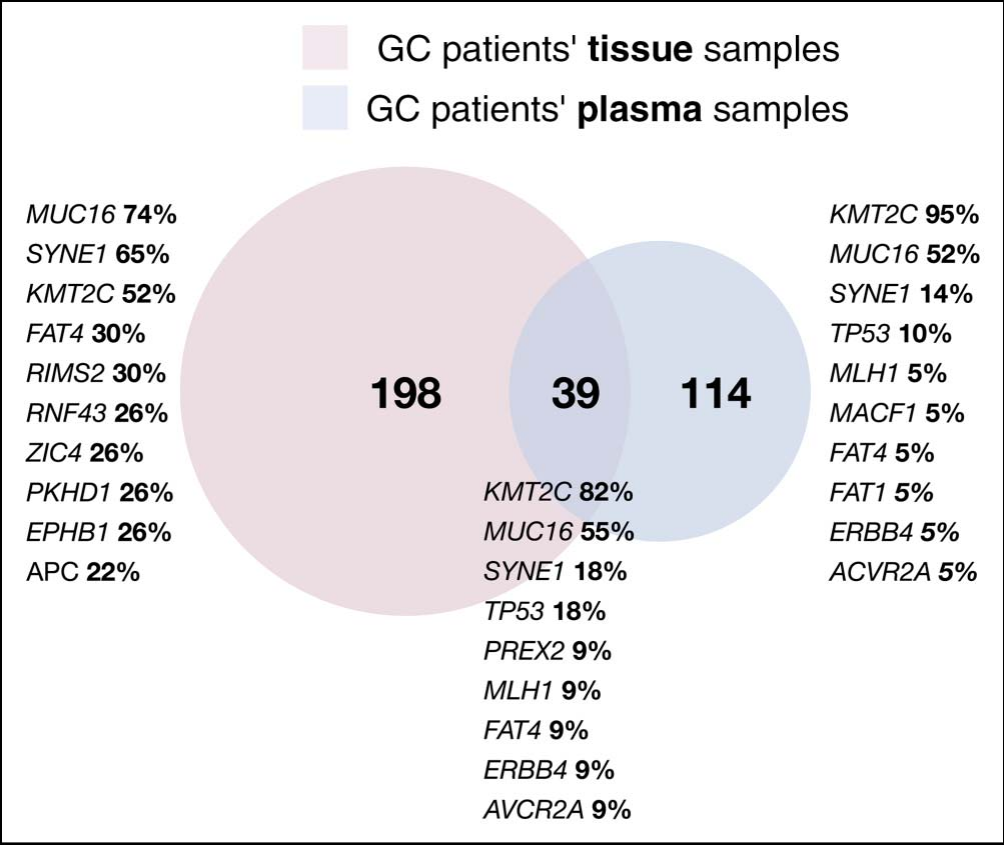
Venn diagram shows the quantity of unique and shared somatic alterations detected in GC patient tissue and plasma samples. Genes listed represents top 10 mutated genes in each case (tissue only, plasma only, and shared) and their frequency. GC, gastric cancer.

Deep sequencing (40,000× raw coverage) of our custom gene panel was performed for plasma cfDNA samples only. Venn diagram shows the number of detected variants in tissue and plasma (Figure 5).

Overall, somatic cfDNA alterations were observed in 21 of 23 patients with alteration-positive GC tissue samples (91.3%) (Figure 5; Supplementary Table 5 [see Supplemental Digital Content 7, http://links.lww.com/CTG/A685]). On average, 5.4 somatic variants per sample were detected in plasma cfDNA of patients with GC (range from 1 to 14). Tissue matching cfDNA alterations were detected in 11 of 23 alteration-positive GC tissue samples (47.8%) (Figure 5). On average, 3.5 tissue matching somatic variants in plasma cfDNA were detected per sample (range from 1 to 12).

**Figure 6.**
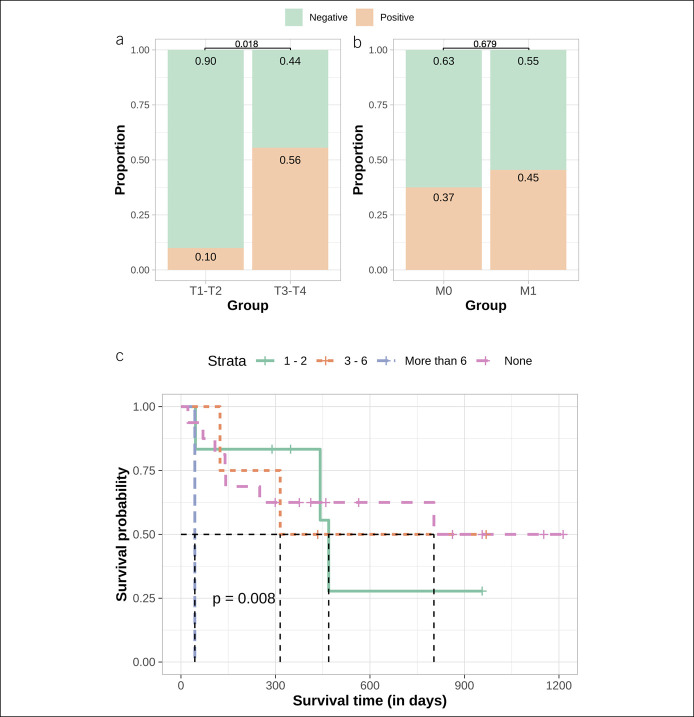
(**a**) Proportion of positive and negative samples with matching tissue and plasma cfDNA alterations comparing groups of T1-T2 and T3-T4. Approximately 10.0% of T1-T2 GC patients and 56.0% of T3-T4 GC patients had a detectable amount of circulating tumor DNA; difference was statistically significant (*P* = 0.018); (**b**) proportion of positive and negative samples with matching tissue and plasma cfDNA alterations comparing groups of M0 and M1. Approximately 37.0% of the GC without distant metastasis and 45.0% of GC with distant metastasis had a detectable amount of tumor cfDNA; difference was not significant (*P* = 0.679); (**c**) Kaplan-Meier survival analysis of patients with GC with 0 (pink line), 1–2 (green line), 3–6 (orange line), or more than 6 (blue line) tissue matching alterations detected in plasma cfDNA. Average survival in days decreases gradually when comparing patients with increasing quantity of mutations (*P* = 0.008). cfDNA, cell-free DNA; GC, gastric cancer.

Next, we compared the quantity of tissue matching somatic variants in plasma cfDNA with GC clinical features and analyzed correlation with total cfDNA yield, serum level of oncoproteins, and age. Concordantly with literature (25,26), the quantity of unique somatic alterations detected in tissue and plasma and the quantity of tissue matching alterations in plasma revealed positive moderate correlation with age (tissue: R = 0.47, *P* = 0.012; plasma: R = 0.4, *P* = 0.035; matching variants: R = 0.38, *P* = 0.048 [see Supplementary Figure 4, Supplemental Digital Content 8, http://links.lww.com/CTG/A686]). Our analysis revealed that cfDNA sequence alterations derived from tumor tissue were detected significantly more often in samples of the patients with larger tumors (T3-T4—55.6% and T1-T2—10.0%, χ^2^ = 5.59, *P* = 0.018) (Figure 6a) and in patients with distal metastasis (not significantly) (45.5% and 37.5%, M1 and M0, respectively, χ^2^ = 0.17, *P* value = 0.679) (Figure 6b). Survival analysis showed that patients without sequence alterations in cfDNA had a median survival time (MST) of 803 days, whereas MST for patients with 1–2 cfDNA sequence alterations was 469 days. MST for patients with 3–6 cfDNA sequence alterations and more than 6 cfDNA sequence alterations was 315 and 44 days, respectively (*P* value = 0.008) (Figure 6c). In addition, Cox proportional hazards model for the survival analysis was used. Model included not only tissue matching somatic variants detected in plasma but also patients' demographics and tumors characteristics: age, gender, and size of the primary tumor based on tumor–node–metastasis staging. Results showed slight gender impact on survival estimation (padj = 0.0410) and significant effect of more than 6 variants detected in plasma (padj = 0.0186) for shorter lifespan.

### Qualitative and quantitative analysis of somatic variants in plasma discriminates patients with distant metastases

The role of multilayer molecular profiling in the discrimination of patients with larger tumors (T3-T4) and distant metastases was evaluated by including analytes such as concentration of oncoproteins CA 19-9, Cancer Antigen 72-4, CEA, MSI status, TMB, quantity of somatic mutations (unique or matching the tumor tissue), presence or absence of somatic mutations (unique or matching the tumor tissue), and specific mutations of the most mutated genes. Our analysis revealed that the quantity of tissue matching variants and the presence of any somatic alteration in plasma cfDNA was shown to be significant for discrimination between M0 and M1 groups (classification analysis resulted in area under curve = 0.744).

## DISCUSSION

In this study, we present a robust analysis of liquid biopsy for GC using circulating plasma cfDNA. We show that somatic mutations determined by WES in GC tissues can be tracked in the blood of patients with GC. Furthermore, our study suggests that qualitative and quantitative analysis of somatic variants in the plasma cfDNA might be a promising approach to discriminate patients with advanced disease.

Raised cfDNA levels were first reported in the serum of patients with cancer in 1977 (27). However, it was shown that concentration of cfDNA could increase because of number of physiological conditions, and more specific analysis of circulating nucleic acids is needed. Circulating tumor DNA can be detected in any body fluids, does not require additional analysis tools such as cell sorting as in the case of circulating tumor cell analysis, and has a very high clinical potential: applications from noninvasive genomic analysis of cancer, quantification of disease burden, disease burden monitoring, and clonal evolution. Despite the recent effort (The Cancer Genome Atlas Research Network) (28), there is still high need for more appropriate gene panels for cfDNA analysis which could be implemented in the routine diagnostics. To analyze wide molecular spectra and investigate genetic alterations in the GC patient group of the European descent, we performed WES for tumor tissue and WBC samples. Twenty-three of 29 patients with GC (79.31%) had cancer-associated somatic alterations detected in tissue. All mutated genes were previously associated with gastric tumorigenesis and reported in the Catalogue Of Somatic Mutations In Cancer database (29). Signaling pathway enrichment analysis revealed that genes which we found to be mutated were involved in Wnt and cadherin pathways (see Supplementary Figure 3, Supplemental Digital Content 9, http://links.lww.com/CTG/A687) (30). Based on our WES results, a custom 38-gene panel for deep targeted sequencing of plasma cfDNA was designed. To the best of our knowledge, this is the first study conducted in patients with GC which implemented UMI error correction and deep sequencing for accurate cfDNA mutational analysis. This approach allowed us to determine somatic alterations in plasma cfDNA samples for 21 of 23 alteration-positive tissue samples (91.3%) and tumor tissue matching alterations for 11 of 23 alteration-positive tissue samples (47.8%). By comparison, previously reported plasma ctDNA mutational concordance with tissue ranged from 33.9% to 58% (8,13,14), and the differences could be explained by GC tissue molecular heterogeneity (31).

Furthermore, we have compared the quantity of tissue matching alterations detected in plasma cfDNA with different clinical features. In concordance to other studies, the analysis has revealed that alterations derived from tumor tissue were detected significantly more often in samples from the patients with more advanced tumors (6,8) and could be associated with worse survival (8). But, our data also indicated that even relatively small GC tumors (10% of T1-T2) could shed detectable amounts of ctDNA into the blood stream. Multicomponent analysis of variable importance based on machine learning algorithms showed that combination of quantity of tissue matching alterations in cfDNA and presence of any somatic alteration in plasma cfDNA was the most accurate when discriminating patients with distal metastasis (area under curve = 0.744). However, it is important to note that more than a third of gastric tumors without distant site metastasis still gave rise to detectable cfDNA molecules carrying somatic alterations. Therefore, we believe that an ability of our custom cfDNA panel to detect even a fraction of patients with nonadvanced tumors (early stages or without metastasis) could improve early cancer detection and increase survival rates (32). Studies report strong correlation between tumor-derived cfDNA detection rates and stage of tumors and in concordant with our findings show that detection rate is around 30% for tumors without distant metastasis (6,8,33,34). Moreover, survival analysis revealed that an increased quantity of somatic mutations in plasma cfDNA is associated with the worse patient's survival. Well-known cancer diagnostic analytes (MSI status, TMB, and oncoproteins) did not reveal any significant impact in our variable importance analysis or our discrimination analysis. These findings support the great need of new minimally invasive molecular markers for GC diagnosis and disease state monitoring.

In addition, we observed that the total cfDNA yield is increased in patients with GC. The higher total cfDNA yield in GC is consistent with previous studies of gastrointestinal cancers (35–38). Although results of various studies show that levels of oncoproteins such as CEA hardly correlate with clinicopathological features (39,40), we found moderate positive correlation with total cfDNA yield and serum CEA levels for patients with GC. The logical explanation for this correlation could be that increased levels of both total cfDNA yield and CEA are observed during tumorigenesis. In the analysis of cfDNA fragment distribution, we showed that the higher total cfDNA yield in GC affected all fragment sizes and mononucleosomal and dinucleosomal fragments were smaller in the patients with GC compared with the control. This observation supports the hypoxia theory: Rapidly growing tumor cells lack oxygen; hypoxia induces necrosis which leads to phagocytosis of tumor cells and DNA fragment release to the blood stream.

The study has some limitations. Study sample size is small; however, study population was well clinically defined and tested for many clinically relevant variables. Healthy controls' plasma cfDNA was not sequenced while healthy controls usually have a very low total cfDNA yield and extremely low ctDNA fraction. This could result to inconsistencies and sequencing errors. Although the gene panel was not evaluated in the independent validation group, all variants were manually checked on integrative genomics viewer. Nevertheless, we believe that this study adds very important new data for the development of clinically relevant liquid biopsy tools in patients with GC.

In conclusion, sequencing-based approaches have the advantage of being flexible and capable of detecting a wide range of aberrations in tumor genomes. Therefore, in this study, WES was performed to analyze the GC tissue mutational profile and to develop a custom panel for cfDNA mutational profile analysis. It is important to note that by using our gene panel and UMI correction, we were able to detect tumor-derived cfDNA even for small tumors and tumors without distant metastasis and identify a solid proportion of patients with GC carrying somatic alterations in plasma cfDNA. We found that the quantity of somatic alterations could be associated with overall patients' survival. Further investigation of plasma cfDNA could implement larger cohorts of the patients with GC and analysis of MAF in cfDNA at different disease time points and/or disease status (e. g. relapse or remission). The implementation of plasma cfDNA analysis into routine cancer testing is still technically challenging, and more population-based screening studies are still needed. However, given the progress in NGS technology and new methods of processing complex data, tumor-derived cfDNA even today shows potential clinical utility as a noninvasive analyte for the characterization of an individual patient's tumor genome.

## CONFLICTS OF INTEREST

**Guarantor of the article**: Jurgita Skieceviciene, PhD.

**Specific author contributions**: Juozas Kupcinskas, MD, PhD, and Jurgita Skieceviciene, PhD, contributed equally to this work. J.S., J.K., and A.F.: supervision and conceptualization. M.F. and G.V.: data collection, data analysis, interpretation, and visualization. G.V. and J.S.: writing original draft. M.F., J.K., A.F., and J.S.: manuscript review and editing. All authors approved the final manuscript version for submission.

**Financial support**: The study is a part of the MULTIOMICS project that has received funding from European Social Fund (project No. 09.3.3-LMT-K-712-01-0130) under grant agreement with the Research Council of Lithuania (LMTLT). M.F. is supported by the Deutsche Forschungsgemeinschaft (DFG).

**Potential competing interests**: The authors declare that they have no conflict of interest.

**Ethics statement**: Informed consent was obtained from all patients. Research was approved by the Kaunas Regional Biomedical Research Ethics Committee (No. BE-2-10, May 8, 2011 and No. BE-2-31, June 5, 2018, Kaunas, Lithuania).

**Study Highlights**

**WHAT IS KNOWN**

✓ gastric cancer (GC) diagnosis in late stages and high mortality rates indicate the need for new molecular tools.
✓ Conventional diagnostic techniques or current molecular biomarkers have a very limited role for early diagnosis of GC.
✓ Tumor-derived DNA (ctDNA) found in plasma of patients with cancer carry genetic information of the tumor which could be assessed by minimally invasive way.

**WHAT IS NEW HERE**

✓ By using GC tissue whole exome sequencing, we developed a custom 38-gene panel and performed cfDNA deep targeted sequencing in plasma samples.
✓ This unique gene panel enabled to identify a solid proportion of patients with GC carrying somatic alterations in plasma cfDNA, including patients whose disease was in early stages.
✓ Multilayer molecular machine learning–based analysis indicated that the quantity of tissue matching variants and the presence of any somatic alteration in plasma cfDNA is significant for discrimination between M0 and M1 groups.

## Supplementary Material

SUPPLEMENTARY MATERIAL
