# Supplementary material for: Liquid Biopsy in Gastric Cancer: Analysis of Somatic Cancer Tissue Mutations in Plasma Cell-Free DNA for Predicting Disease State and Patient Survival
Source: Clin Transl Gastroenterol. 2021 Sep 24;12(9):e00403. doi: 10.14309/ctg.0000000000000403 (PMC8462609; doi:10.14309/ctg.0000000000000403)
Supplement: SUPPLEMENTARY MATERIAL [file ct9-12-e00403-s001.pdf]

Supplementary table 1. Patient information

Sample	TNM	Differentiation grade	Type	Lauren classification	TMB*	Gender	Age	MSI status**	CA19.9	CEA	CA72.4
I00650	T1b, N2, M0	G3	Adenocarcinoma	Intestinal	95.128	M	66	Positive	-	803.73	0.60
I00633	T1b, N2, M0	G2	Papillary adenocarcinoma	Intestinal	13.846	M	75	Negative	-	437.91	1.07
I00639	T1b, N0, M0	G2	Adenocarcinoma	Intestinal	8.205	M	81	Negative	-	295.65	1.03
I00641	Tx, Nx, M1	G3	Carcinoma	Diffuse	11.026	M	72	Negative	354.54	734.14	3.59
I00646	T2, N1, M0	G3	Adenocarcinoma	Diffuse	9.231	M	51	Negative	-	969.55	1.40
I00631	T2, N0, M0	G3	Adenocarcinoma	Diffuse	13.846	F	43	Negative	-	508.45	2.29
I00630	T2, N3a, M0	G3	Adenocarcinoma	Indeterminate	58.462	M	86	Negative	138.99	611.71	0.96
I00654	T2, N3, M1	G3	Adenocarcinoma	Diffuse	12.821	M	75	Negative	-	568.60	0.77
I00634	T2, N0, M0	G2	Adenocarcinoma	Intestinal	10.769	M	63	Negative	-	438.41	0.51
I00640	T2, N0, M1	unknown	Adenocarcinoma	Diffuse	10.513	F	42	Negative	-	577.59	0.62
I00649	T3, N0, M0	G2	Adenocarcinoma	Intestinal	10.769	M	55	Negative	-	546.86	0.64
I00647	T3, N3, M1	G3	Adenocarcinoma	Indeterminate	67.179	M	85	Positive	-	825.42	0.73
I00655	T3, N2, M1	G2	Adenocarcinoma	Intestinal	7.436	M	53	Negative	-	656.27	0.48
I00632	T3, N3a, M0	G2	Adenocarcinoma	Intestinal	97.436	M	63	Positive	-	409.79	1.16
I00644	T3, N2, M1	G2	Adenocarcinoma	Intestinal	10.769	F	77	Negative	-	565.33	1.13
I00643	T3, N1, M0	unknown	Carcinoma	unknown	12.308	F	72	Negative	-	666.97	1.23
I00638	T3, N0, M0	unknown	Signet ring cell carcinoma	Diffuse	11.026	M	70	Negative	-	335.73	0.55
I00635	T3, N2, M0	G2	Adenocarcinoma	Diffuse	8.462	M	72	Negative	-	365.24	4.36
I00653	T4a, N3b, M0	G3	Adenocarcinoma	Intestinal	20.256	F	77	Negative	49.83	964.26	1.15
I00642	T4, N1, Mx	G3	Adenocarcinoma	Diffuse	14.359	M	62	Negative	-	379.02	1.31
I00658	T4a, N2, M0	G2	adenocarcinoma	Intestinal	13.590	M	81	Negative	118.51	574.91	1.14
I00651	T4a, N3a, M1	G3	Adenocarcinoma	Intestinal	12.821	M	80	Negative	-	596.86	0.98
I00652	T4b, N3b, M0	G2	Adenocarcinoma	Intestinal	12.564	M	67	Negative	10.41	689.79	0.88
I00645	T4, N1, M1	G2	Adenocarcinoma	Intestinal	12.564	M	72	Negative	-	571.65	0.58
I00636	T4, N3, M1	G2	Adenocarcinoma	Intestinal	14.103	F	77	Negative	-	242.37	1.43
I00657	T4b, N3, M1	G3	Adenocarcinoma	Diffuse	78.462	M	85	Negative	-	342.80	0.56
I00656	T4b, N3b, M1	G3	Adenocarcinoma	Diffuse	11.795	F	66	Negative	30.41	536.01	0.77
I00629	unknown	unknown	Adenocarcinoma	unknown	9.744	M	65	Negative	-	432.97	0.89

* Tumor mutational burden (the number of somatic mutations in the coding region per megabase (Mb), including SNVs and small INDELs)
** Microsatellite instability from WES data was evaluated using MSIsensor (Niu B, et al. *Bioinformatics* , 2014).
