# Supplementary material for: Liquid Biopsy in Gastric Cancer: Analysis of Somatic Cancer Tissue Mutations in Plasma Cell-Free DNA for Predicting Disease State and Patient Survival
Source: Clin Transl Gastroenterol. 2021 Sep 24;12(9):e00403. doi: 10.14309/ctg.0000000000000403 (PMC8462609; doi:10.14309/ctg.0000000000000403)
Supplement: SUPPLEMENTARY MATERIAL [file ct9-12-e00403-s002.pdf]

Supplementary table 2. Genes covered by custom targeted NGS panel

ACVR2A	CDH1	ERBB4	GLI3	MSH6	PREX2	SYNE1	TRRAP
APC	CTNNB1	FAT1	KMT2C	MUC16	PTEN	SMAD4	ZIC4
ARID1A	EGFR	FAT4	KRAS	PBRM1	RHOA	SPEN	TTN
ATM	EPHB1	FBXW7	MACF1	PIK3CA	RIMS2	STK11	
CCND1	ERBB2	FHIT	MLH1	PKHD1	RNF43	TP53	
