# Supplementary material for: Liquid Biopsy in Gastric Cancer: Analysis of Somatic Cancer Tissue Mutations in Plasma Cell-Free DNA for Predicting Disease State and Patient Survival
Source: Clin Transl Gastroenterol. 2021 Sep 24;12(9):e00403. doi: 10.14309/ctg.0000000000000403 (PMC8462609; doi:10.14309/ctg.0000000000000403)
Supplement: SUPPLEMENTARY MATERIAL [file ct9-12-e00403-s003.pdf]

Supplementary table 3. Sequencing metrics

Source	Sample name	Read length	Reads	Target territory	On target bases	Portion target covered 10x* or 100x† (%)	Mean target coverage
Tumour tissue	**I00629**	100	40282864	38874504	2534031537	98.10%	65
Tumour tissue	**I00630**	100	31793768	38874504	1923669778	97.20%	49
Tumour tissue	**I00631**	100	39598528	38874504	2328821716	96.80%	60
Tumour tissue	**I00632**	100	44277250	38874504	2712705244	98.40%	70
Tumour tissue	**I00633**	100	52243412	38874504	3267217748	98.60%	84
Tumour tissue	**I00634**	100	41528506	38874504	2602067809	98.00%	67
Tumour tissue	**I00635**	100	29021988	38874504	1805218890	97.10%	46
Tumour tissue	**I00636**	100	57057806	38874504	3505903349	98.20%	90
Tumour tissue	**I00637**	100	66056844	38874504	4019258359	98.60%	103
Tumour tissue	**I00638**	100	52748112	38874504	3185813494	98.50%	82
Tumour tissue	**I00639**	100	47417780	38874504	2868093696	98.40%	74
Tumour tissue	**I00640**	100	59901720	38874504	3680985270	98.70%	95
Tumour tissue	**I00641**	100	54574638	38874504	3341487397	98.60%	86
Tumour tissue	**I00642**	100	48512136	38874504	2978437474	98.60%	77
Tumour tissue	**I00643**	100	49245172	38874504	2993808093	98.50%	77
Tumour tissue	**I00644**	100	52252224	38874504	3251594301	98.40%	84
Tumour tissue	**I00645**	100	51955968	38874504	3171732979	98.60%	82
Tumour tissue	**I00646**	100	49152368	38874504	2973362959	98.50%	76
Tumour tissue	**I00647**	100	47537862	38874504	2918408751	98.30%	75
Tumour tissue	**I00648**	100	35807900	38874504	2194361622	98.00%	56
Tumour tissue	**I00649**	100	42305166	38874504	2595176666	98.20%	67
Tumour tissue	**I00650**	100	36842672	38874504	2270969470	97.80%	58
Tumour tissue	**I00651**	100	45690082	38874504	2825309984	98.40%	73
Tumour tissue	**I00652**	100	50803038	38874504	3035561975	98.50%	78
Tumour tissue	**I00653**	100	60651446	38874504	3806432054	98.50%	98
Tumour tissue	**I00654**	100	42497178	38874504	2586339479	98.40%	67
Tumour tissue	**I00655**	100	38664244	38874504	2367687329	98.10%	61
Tumour tissue	**I00656**	100	51980522	38874504	3201771097	98.60%	82
Tumour tissue	**I00657**	100	48364582	38874504	2929082393	98.40%	75
Tumour tissue	**I00658**	100	43693546	38874504	2686808383	98.30%	69
Normal WBC	**I00629**	100	56456258	38874504	3495483961	98.60%	90
Normal WBC	**I00630**	100	47463474	38874504	2954279668	98.50%	76
Normal WBC	**I00631**	100	53920518	38874504	3335393834	98.60%	86
Normal WBC	**I00632**	100	38211594	38874504	2228233776	97.80%	57
Normal WBC	**I00633**	100	52163908	38874504	3273091119	98.60%	84
Normal WBC	**I00634**	100	45216158	38874504	2871445225	98.50%	74
Normal WBC	**I00635**	100	43097730	38874504	2657192522	98.10%	68
Normal WBC	**I00636**	100	66800050	38874504	4114774092	98.50%	106
Normal WBC	**I00637**	100	43289140	38874504	2653395975	98.30%	68
Normal WBC	**I00638**	100	46504658	38874504	2845735052	98.40%	73
Normal WBC	**I00639**	100	48598028	38874504	2935161434	98.50%	76
Normal WBC	**I00640**	100	51267144	38874504	3066913932	98.60%	79
Normal WBC	**I00641**	100	43191772	38874504	2624641535	98.30%	68
Normal WBC	**I00642**	100	44977858	38874504	2770424349	98.40%	71

Normal WBC	**I00643**	100	48426042	38874504	2931406618	98.40%	75
Normal WBC	**I00644**	100	45151842	38874504	2784669586	98.40%	72
Normal WBC	**I00645**	100	49024916	38874504	2974346165	98.50%	77
Normal WBC	**I00646**	100	52248042	38874504	3182211080	98.60%	82
Normal WBC	**I00647**	100	62326708	38874504	3781696050	98.70%	97
Normal WBC	**I00648**	100	51640638	38874504	3188232921	98.60%	82
Normal WBC	**I00649**	100	44743838	38874504	2757079391	98.40%	71
Normal WBC	**I00650**	100	53467836	38874504	3271383055	98.60%	84
Normal WBC	**I00651**	100	44500884	38874504	2715131453	98.20%	70
Normal WBC	**I00652**	100	45628850	38874504	2820255777	98.40%	73
Normal WBC	**I00653**	100	40348852	38874504	2481130499	98.20%	64
Normal WBC	**I00654**	100	43352030	38874504	2671553880	98.40%	69
Normal WBC	**I00655**	100	38446290	38874504	2340708622	98.00%	60
Normal WBC	**I00656**	100	48637704	38874504	2992454223	98.50%	77
Normal WBC	**I00657**	100	54253562	38874504	3301333816	98.50%	85
Normal WBC	**I00658**	100	41176270	38874504	2477256642	98.10%	64
Plasma cfDNA	**I00629**	150	1925440020	389683	96078373757	99.80%	246555
Plasma cfDNA	**I00630**	150	116677368	389683	3194464607	99.70%	8198
Plasma cfDNA	**I00631**	150	88608160	389683	2834064800	99.80%	7273
Plasma cfDNA	**I00632**	150	526775610	389683	25420655512	99.80%	65234
Plasma cfDNA	**I00633**	150	588213838	389683	27556496287	99.80%	70715
Plasma cfDNA	**I00634**	150	54063944	389683	1296533761	99.70%	3327
Plasma cfDNA	**I00636**	150	149282498	389683	6228095221	99.80%	15982
Plasma cfDNA	**I00637**	150	215892490	389683	5943799631	99.70%	15253
Plasma cfDNA	**I00638**	150	213537244	389683	9676295448	99.90%	24831
Plasma cfDNA	**I00639**	150	89747974	389683	2243659177	99.60%	5758
Plasma cfDNA	**I00642**	150	943467732	389683	46908613955	99.80%	120376
Plasma cfDNA	**I00643**	150	363844884	389683	17479661439	99.90%	44856
Plasma cfDNA	**I00645**	150	120693718	389683	10640482906	99.80%	27305
Plasma cfDNA	**I00647**	150	113182792	389683	3216035249	99.70%	8253
Plasma cfDNA	**I00648**	150	89996208	389683	2526096799	99.70%	6482
Plasma cfDNA	**I00649**	150	258635882	389683	11959911073	99.80%	30691
Plasma cfDNA	**I00650**	150	69343174	389683	1575012955	99.60%	4042
Plasma cfDNA	**I00651**	150	128449582	389683	3458995901	99.70%	8876
Plasma cfDNA	**I00652**	150	98132034	389683	2552281293	99.70%	6550
Plasma cfDNA	**I00653**	150	93471852	389683	2536023493	99.70%	6508
Plasma cfDNA	**I00654**	150	131092308	389683	3532523097	99.80%	9065
Plasma cfDNA	**I00657**	150	165396142	389683	6991019337	99.80%	17940
Plasma cfDNA	**I00658**	150	201540666	389683	8775071375	99.80%	22518

* - for tumour tissue and matching normal WBC samples; † - for plasma cfDNA samples.
