# Supplementary material for: Liquid Biopsy in Gastric Cancer: Analysis of Somatic Cancer Tissue Mutations in Plasma Cell-Free DNA for Predicting Disease State and Patient Survival
Source: Clin Transl Gastroenterol. 2021 Sep 24;12(9):e00403. doi: 10.14309/ctg.0000000000000403 (PMC8462609; doi:10.14309/ctg.0000000000000403)
Supplement: SUPPLEMENTARY MATERIAL [file ct9-12-e00403-s004.pdf]

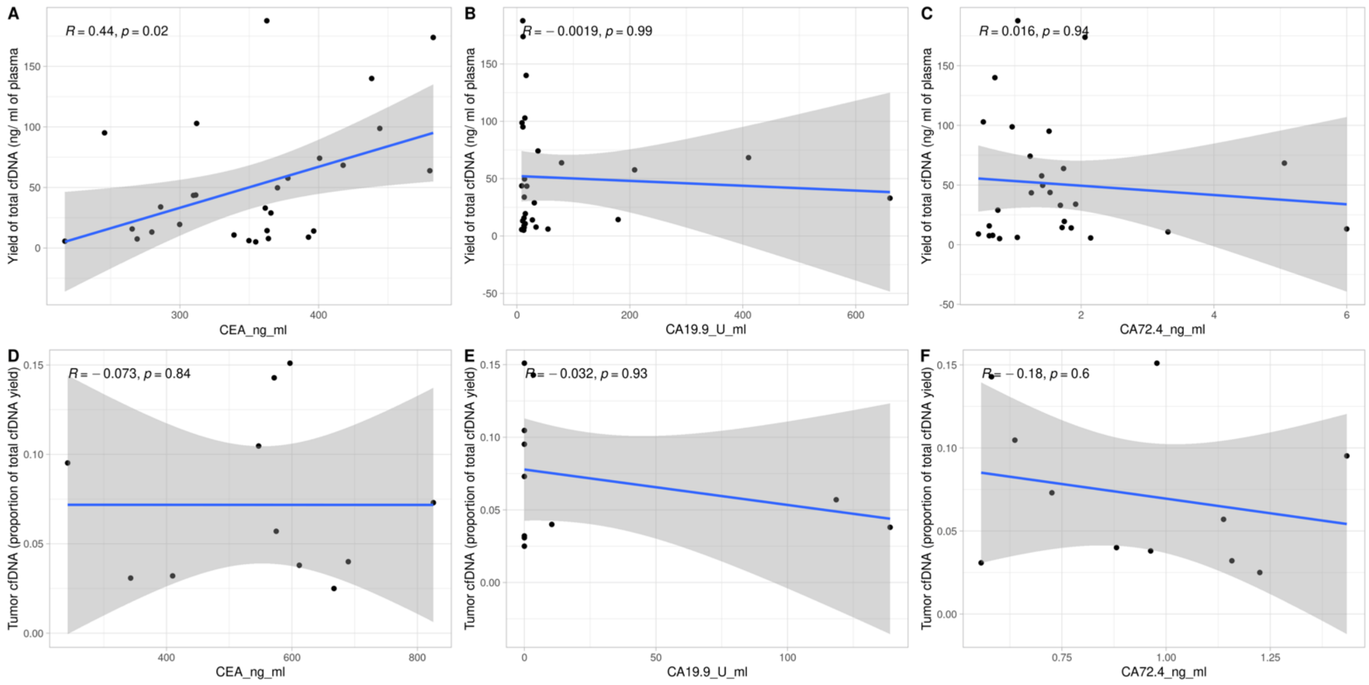

Supplementary Figure 1. Spearman's correlation analysis between level of oncoproteins CEA, CA 19-9, CA 72-4 (x-axis), and total circulating cfDNA (**a-c**) and tumor derived cfDNA yield (**d-f**). Moderate positive correlation was determined between levels of CEA (ng/ml) and total cfDNA yield (R = 0.44, p= 0.02)
