# Supplementary material for: Liquid Biopsy in Gastric Cancer: Analysis of Somatic Cancer Tissue Mutations in Plasma Cell-Free DNA for Predicting Disease State and Patient Survival
Source: Clin Transl Gastroenterol. 2021 Sep 24;12(9):e00403. doi: 10.14309/ctg.0000000000000403 (PMC8462609; doi:10.14309/ctg.0000000000000403)
Supplement: SUPPLEMENTARY MATERIAL [file ct9-12-e00403-s005.pdf]

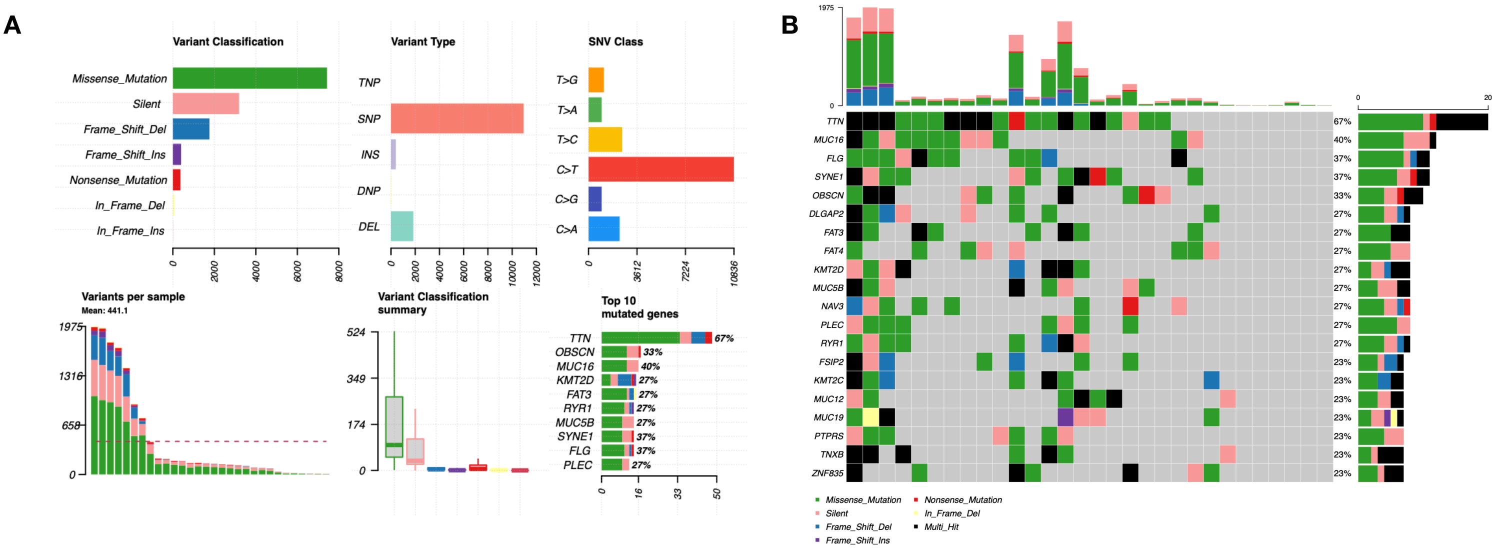

Supplementary Figure 2. Summary of mutational spectra (whole exome sequencing) in GC tissue samples**. a** Summary of mutational spectra (whole exome sequencing) in GC tissue samples including variant classification, variant type, SNV class, variants per sample, variants classification summary and top 10 mutated genes; **b** oncoplot of mutated genes in GC tissue samples
