# Supplementary material for: Liquid Biopsy in Gastric Cancer: Analysis of Somatic Cancer Tissue Mutations in Plasma Cell-Free DNA for Predicting Disease State and Patient Survival
Source: Clin Transl Gastroenterol. 2021 Sep 24;12(9):e00403. doi: 10.14309/ctg.0000000000000403 (PMC8462609; doi:10.14309/ctg.0000000000000403)
Supplement: SUPPLEMENTARY MATERIAL [file ct9-12-e00403-s006.pdf]

Supplementary table 4. Somatic mutations in GC tissue samples

Sample name	Gene	cHGVS	pHGVS	Transcript	Variant classification	Chr	Start	End	REF	ALT
**I00629**	MUC16	c.7248C>T	p.Pro2416Pro	NM_024690.2	Silent	19	9084567	9084567	G	A
**I00629**	FAT4	c.1114C>G	p.Gln372Glu	NM_001291303.1	Missense_Mutation	4	126238680	126238680	C	G
**I00629**	SYNE1	c.11533C>A	p.Pro3845Thr	NM_182961.3	Missense_Mutation	6	152673209	152673209	G	T
**I00629**	GLI3	c.2531T>A	p.Leu844His	NM_000168.5	Missense_Mutation	7	42006140	42006140	A	T
**I00630**	RNF43	c.1976delG	p.Gly659fs	NM_001305544.1	Frame_Shift_Del	17	56435160	56435161	AC	-
**I00630**	MUC16	c.40663_40664delCCinsGT	p.Pro13555Val	NM_024690.2	Missense_Mutation	19	8999511	8999511	G	A
**I00630**	ZIC4	c.1018C>T	p.Arg340Cys	NM_001168378.1	Missense_Mutation	3	147108854	147108854	G	A
**I00630**	PBRM1	c.2223G>A	p.Pro741Pro	NM_018313.4	Silent	3	52643673	52643673	C	T
**I00630**	PKHD1	c.*280C>T		NM_170724.2	3'UTR	6	51586504	51586504	G	A
**I00630**	KMT2C	c.6341G>A	p.Arg2114Lys	NM_170606.2	Missense_Mutation	7	151878604	151878604	C	T
**I00630**	KMT2C	c.3214C>T	p.Gln1072*	NM_170606.2	Nonsense_Mutation	7	151921209	151921209	G	A
**I00630**	KMT2C	c.1012+76G>T		NM_170606.2	Intron	7	151970714	151970714	C	A
**I00630**	TRRAP	c.5917-8G>A		NM_001244580.1	Intron	7	98553761	98553761	G	A
**I00631**	FAT4	c.822G>A	p.Ala274Ala	NM_001291303.1	Silent	4	126238388	126238388	G	A
**I00631**	APC	c.1409-60T>A		NM_000038.5	Intron	5	112162745	112162745	T	A
**I00631**	KMT2C	c.8479_8480delCT	p.Leu2827fs	NM_170606.2	Frame_Shift_Del	7	151874057	151874059	CAG	-
**I00632**	MACF1	c.2785G>A	p.Ala929Thr	NM_012090.5	Missense_Mutation	1	39768731	39768731	G	A
**I00632**	MACF1	c.10567G>A	p.Val3523Ile	NM_012090.5	Missense_Mutation	1	39893909	39893909	G	A
**I00632**	ERBB2	c.648G>A	p.Thr216Thr	NM_004448.3	Silent	17	37866343	37866343	G	A
**I00632**	ERBB2	c.*54G>A		NM_001289938.1	3'UTR	17	37873791	37873791	G	A
**I00632**	RNF43	c.1976delG	p.Gly659fs	NM_001305544.1	Frame_Shift_Del	17	56435160	56435161	AC	-
**I00632**	MUC16	c.28570G>A	p.Glu9524Lys	NM_024690.2	Missense_Mutation	19	9058876	9058876	C	T
**I00632**	MUC16	c.15739A>C	p.Thr5247Pro	NM_024690.2	Missense_Mutation	19	9071707	9071707	T	G
**I00632**	ACVR2A	c.1310delA	p.Lys437fs	NM_001278579.1	Frame_Shift_Del	2	148683685	148683686	TA	-
**I00632**	EPHB1	c.1733A>G	p.Lys578Arg	NM_004441.4	Missense_Mutation	3	134885822	134885822	A	G
**I00632**	APC	c.3271C>T	p.Pro1091Ser	NM_000038.5	Missense_Mutation	5	112174562	112174562	C	T
**I00632**	APC	c.7756dupA	p.Ser2586fs	NM_000038.5	Frame_Shift_Ins	5	112179042	112179043	-	CA
**I00632**	SYNE1	c.10145+120_10145+121insCCCTTCCT		NM_182961.3	Intron	6	152685861	152685869	-	AAGGAAGGG
**I00632**	SYNE1	c.8361G>A	p.Thr2787Thr	NM_182961.3	Silent	6	152708333	152708333	C	T
**I00632**	PKHD1	c.5236+22G>T		NM_138694.3	Intron	6	51889350	51889350	C	A
**I00632**	KMT2C	c.10751G>T	p.Gly3584Val	NM_170606.2	Missense_Mutation	7	151859911	151859911	C	A
**I00632**	KMT2C	c.5183G>A	p.Ser1728Asn	NM_170606.2	Missense_Mutation	7	151880141	151880141	C	T
**I00632**	KMT2C	c.1012+13C>T		NM_170606.2	Intron	7	151970777	151970777	G	A
**I00632**	KMT2C	c.850-17T>G		NM_170606.2	Intron	7	151970969	151970969	A	C
**I00632**	GLI3	c.3098delC	p.Pro1033fs	NM_000168.5	Frame_Shift_Del	7	42005572	42005573	CG	-
**I00632**	GLI3	c.1627G>A	p.Glu543Lys	NM_000168.5	Missense_Mutation	7	42018218	42018218	C	T
**I00632**	GLI3	c.874C>T	p.Arg292Cys	NM_000168.5	Missense_Mutation	7	42079791	42079791	G	A
**I00632**	PREX2	c.2907A>G	p.Gln969Gln	NM_025170.5	Silent	8	69017564	69017564	A	G
**I00633**	RNF43	c.605T>A	p.Leu202Gln	NM_001305544.1	Missense_Mutation	17	56439987	56439987	A	T
**I00633**	STK11	c.595G>T	p.Glu199*	NM_000455.4	Nonsense_Mutation	19	1220502	1220502	G	T
**I00633**	MUC16	c.13179T>C	p.Pro4393Pro	NM_024690.2	Silent	19	9074267	9074267	A	G
**I00633**	ZIC4	c.839-918G>A		NM_001168378.1	Intron	3	147109951	147109951	C	T
**I00633**	FAT4	c.12852C>T	p.Ser4284Ser	NM_001291303.1	Silent	4	126408529	126408529	C	T
**I00633**	SYNE1	c.15293+88G>A		NM_182961.3	Intron	6	152647343	152647343	C	T
**I00633**	RIMS2	c.922A>G	p.Ile308Val	NM_001100117.2	Missense_Mutation	8	104897749	104897749	A	G
**I00634**	MUC16	c.11969C>A	p.Ala3990Glu	NM_024690.2	Missense_Mutation	19	9075477	9075477	G	T
**I00634**	FAT4	c.12110T>G	p.Leu4037Arg	NM_001291303.1	Missense_Mutation	4	126389871	126389871	T	G
**I00636**	SPEN	c.1697A>C	p.Gln566Pro	NM_015001.2	Missense_Mutation	1	16247426	16247426	A	C
**I00636**	MUC16	c.40663_40664delCCinsGT	p.Pro13555Val	NM_024690.2	Missense_Mutation	19	8999511	8999511	G	A
**I00636**	MUC16	c.40644T>C	p.Asp13548Asp	NM_024690.2	Silent	19	8999531	8999531	A	G
**I00636**	KMT2C	c.2968A>G	p.Ser990Gly	NM_170606.2	Missense_Mutation	7	151927016	151927016	T	C
**I00636**	KMT2C	c.1005T>A	p.Pro335Pro	NM_170606.2	Silent	7	151970797	151970797	A	T
**I00636**	KMT2C	c.925C>T	p.Pro309Ser	NM_170606.2	Missense_Mutation	7	151970877	151970877	G	A
**I00639**	APC	c.784G>T	p.Glu262*	NM_000038.5	Nonsense_Mutation	5	112137030	112137030	G	T
**I00639**	SYNE1	c.2797G>T	p.Glu933*	NM_182961.3	Nonsense_Mutation	6	152776656	152776656	C	A
**I00642**	MUC16	c.32425C>A	p.Arg10809Arg	NM_024690.2	Silent	19	9049206	9049206	G	T

**I00642**	APC	c.4666dupA	p.Thr1556fs	NM_000038.5	Frame_Shift_Ins	5	112175951	112175952	-	GA
**I00642**	PKHD1	c.10604A>C	p.Tyr3535Ser	NM_138694.3	Missense_Mutation	6	51524320	51524320	T	G
**I00642**	EGFR	c.2380C>T	p.Pro794Ser	NM_005228.3	Missense_Mutation	7	55249082	55249082	C	T
**I00642**	RIMS2	c.1625-17T>G		NM_001100117.2	Intron	8	104922345	104922345	T	G
**I00643**	MUC16	c.40611-16A>C		NM_024690.2	Intron	19	8999580	8999580	T	G
**I00644**	SYNE1	c.24642+47A>G		NM_182961.3	Intron	6	152470565	152470565	T	C
**I00644**	PKHD1	c.3729G>A	p.Thr1243Thr	NM_138694.3	Silent	6	51890879	51890879	C	T
**I00644**	PREX2	c.*4T>G		NM_025170.5	3'UTR	8	69017601	69017601	T	G
**I00645**	TP53	c.467delG	p.Arg156fs	NM_000546.5	Frame_Shift_Del	17	7578462	7578463	GC	-
**I00645**	MUC16	c.40734C>T	p.Ile13578Ile	NM_024690.2	Silent	19	8999441	8999441	G	A
**I00645**	MUC16	c.40732A>G	p.Ile13578Val	NM_024690.2	Missense_Mutation	19	8999443	8999443	T	C
**I00645**	MUC16	c.40713G>A	p.Leu13571Leu	NM_024690.2	Silent	19	8999462	8999462	C	T
**I00645**	MUC16	c.40707G>C	p.Trp13569Cys	NM_024690.2	Missense_Mutation	19	8999468	8999468	C	G
**I00645**	SYNE1	c.18012+58G>T		NM_182961.3	Intron	6	152614665	152614665	C	A
**I00645**	SYNE1	c.17635T>G	p.Cys5879Gly	NM_182961.3	Missense_Mutation	6	152621823	152621823	A	C
**I00645**	KMT2C	c.1012+76G>T		NM_170606.2	Intron	7	151970714	151970714	C	A
**I00646**	MUC16	c.20153T>A	p.Ile6718Asn	NM_024690.2	Missense_Mutation	19	9067293	9067293	A	T
**I00646**	SYNE1	c.15336G>T	p.Glu5112Asp	NM_182961.3	Missense_Mutation	6	152647195	152647195	C	A
**I00646**	RIMS2	c.1288A>G	p.Asn430Asp	NM_001100117.2	Missense_Mutation	8	104898115	104898115	A	G
**I00647**	ARID1A	c.2296delC	p.Gln766fs	NM_006015.4	Frame_Shift_Del	1	27088681	27088682	TC	-
**I00647**	MACF1	c.5765G>A	p.Arg1922Gln	NM_012090.5	Missense_Mutation	1	39824376	39824376	G	A
**I00647**	ATM	c.4110-33C>T		NM_000051.3	Intron	11	108159671	108159671	C	T
**I00647**	RNF43	c.469C>T	p.Leu157Leu	NM_001305544.1	Silent	17	56440749	56440749	G	A
**I00647**	EPHB1	c.2025C>T	p.Phe675Phe	NM_004441.4	Silent	3	134911560	134911560	C	T
**I00647**	ZIC4	c.839-791G>A		NM_001168378.1	Intron	3	147109824	147109824	C	T
**I00647**	ZIC4	c.839-812C>A		NM_001168378.1	Intron	3	147109845	147109845	G	T
**I00647**	MLH1	c.37G>A	p.Glu13Lys	NM_000249.3	Missense_Mutation	3	37035075	37035075	G	A
**I00647**	FAT4	c.7T>C	p.Leu3Leu	NM_001291303.1	Silent	4	126237573	126237573	T	C
**I00647**	SYNE1	c.15438+15C>T		NM_182961.3	Intron	6	152647078	152647078	G	A
**I00647**	SYNE1	c.9408C>T	p.Thr3136Thr	NM_182961.3	Silent	6	152694271	152694271	G	A
**I00647**	PKHD1	c.3804C>T	p.Ala1268Ala	NM_138694.3	Silent	6	51890804	51890804	G	A
**I00647**	KMT2C	c.14404G>A	p.Gly4802Arg	NM_170606.2	Missense_Mutation	7	151836816	151836816	C	T
**I00647**	KMT2C	c.3323+62delA		NM_170606.2	Intron	7	151921037	151921037	GT	G
**I00647**	KMT2C	c.1012+103T>G		NM_170606.2	Intron	7	151970687	151970687	A	C
**I00647**	GLI3	c.3556C>T	p.Pro1186Ser	NM_000168.5	Missense_Mutation	7	42005115	42005115	G	A
**I00647**	GLI3	c.2234A>C	p.Asp745Ala	NM_000168.5	Missense_Mutation	7	42007391	42007391	T	G
**I00647**	TRRAP	c.1160G>A	p.Arg387His	NM_001244580.1	Missense_Mutation	7	98506395	98506395	G	A
**I00647**	TRRAP	c.3508C>T	p.Arg1170Trp	NM_001244580.1	Missense_Mutation	7	98528370	98528370	C	T
**I00647**	TRRAP	c.11328C>T	p.Asp3776Asp	NM_001244580.1	Silent	7	98609726	98609726	C	T
**I00647**	RIMS2	c.592G>C	p.Val198Leu	NM_001100117.2	Missense_Mutation	8	104778659	104778659	G	C
**I00647**	PREX2	c.141+79G>A		NM_024870.3	Intron	8	68864849	68864849	G	A
**I00647**	PREX2	c.1610T>C	p.Val537Ala	NM_024870.3	Missense_Mutation	8	68989672	68989672	T	C
**I00648**	ARID1A	c.437delC	p.Pro146fs	NM_006015.4	Frame_Shift_Del	1	27023326	27023327	GC	-
**I00648**	ARID1A	c.2402delG	p.Gly801fs	NM_006015.4	Frame_Shift_Del	1	27088787	27088788	AG	-
**I00648**	ARID1A	c.5000C>T	p.Pro1667Leu	NM_006015.4	Missense_Mutation	1	27102074	27102074	C	T
**I00648**	MACF1	c.1696C>T	p.Arg566*	NM_012090.5	Nonsense_Mutation	1	39753130	39753130	C	T
**I00648**	CCND1	c.683G>A	p.Arg228His	NM_053056.2	Missense_Mutation	11	69462870	69462870	G	A
**I00648**	ERBB2	c.1022-118T>C		NM_004448.3	Intron	17	37868457	37868457	T	C
**I00648**	RNF43	c.1976delG	p.Gly659fs	NM_001305544.1	Frame_Shift_Del	17	56435160	56435161	AC	-
**I00648**	STK11	c.1108+1409G>A		NM_000455.4	Intron	19	1224580	1224580	G	A
**I00648**	MUC16	c.34881A>G	p.Ile11627Met	NM_024690.2	Missense_Mutation	19	9046750	9046750	T	C
**I00648**	MUC16	c.19605G>A	p.Pro6535Pro	NM_024690.2	Silent	19	9067841	9067841	C	T
**I00648**	ZIC4	c.839-1119C>T		NM_001168378.1	Intron	3	147110152	147110152	G	A
**I00648**	CTNNB1	c.1255T>C	p.Cys419Arg	NM_001098209.1	Missense_Mutation	3	41275089	41275089	T	C
**I00648**	PBRM1	c.1726delC	p.Arg576fs	NM_018313.4	Frame_Shift_Del	3	52651369	52651370	CG	-
**I00648**	FAT4	c.536G>A	p.Gly179Asp	NM_001291303.1	Missense_Mutation	4	126238102	126238102	G	A
**I00648**	FAT1	c.2440G>A	p.Asp814Asn	NM_005245.3	Missense_Mutation	4	187628542	187628542	C	T
**I00648**	SYNE1	c.8276C>A	p.Pro2759His	NM_182961.3	Missense_Mutation	6	152708418	152708418	G	T
**I00648**	SYNE1	c.2091C>T	p.Val697Val	NM_182961.3	Silent	6	152784494	152784494	G	A
**I00648**	KMT2C	c.10683T>G	p.Pro3561Pro	NM_170606.2	Silent	7	151859979	151859979	A	C
**I00648**	RIMS2	c.99delT	p.Phe33fs	NM_001282881.1	Frame_Shift_Del	8	104831829	104831830	CT	-
**I00648**	PREX2	c.*117A>G		NM_025170.5	3'UTR	8	69017714	69017714	A	G
**I00649**	TP53	c.586C>T	p.Arg196*	NM_000546.5	Nonsense_Mutation	17	7578263	7578263	G	A
**I00649**	ACVR2A	c.886A>G	p.Met296Val	NM_001278579.1	Missense_Mutation	2	148676085	148676085	A	G
**I00649**	KMT2C	c.2976+31T>C		NM_170606.2	Intron	7	151926977	151926977	A	G
**I00649**	KMT2C	c.1012+80G>T		NM_170606.2	Intron	7	151970710	151970710	C	A

**I00649**	KMT2C	c.850-63C>T		NM_170606.2	Intron	7	151971015	151971015	G	A
**I00649**	PREX2	c.4775+31G>A		NM_024870.3	Intron	8	69136892	69136892	G	A
**I00650**	SPEN	c.6313delG	p.Ala2105fs	NM_015001.2	Frame_Shift_Del	1	16259042	16259043	AG	-
**I00650**	ARID1A	c.437delC	p.Pro146fs	NM_006015.4	Frame_Shift_Del	1	27023326	27023327	GC	-
**I00650**	ARID1A	c.1015delG	p.Ala339fs	NM_006015.4	Frame_Shift_Del	1	27023903	27023904	TG	-
**I00650**	ARID1A	c.2420-84A>G		NM_006015.4	Intron	1	27089380	27089380	A	G
**I00650**	MACF1	c.4629+5595G>A		NM_012090.5	Intron	1	39798620	39798620	G	A
**I00650**	ATM	c.8922G>A	p.Pro2974Pro	NM_000051.3	Silent	11	108235880	108235880	G	A
**I00650**	RNF43	c.1976delG	p.Gly659fs	NM_001305544.1	Frame_Shift_Del	17	56435160	56435161	AC	-
**I00650**	TP53	c.637C>T	p.Arg213*	NM_000546.5	Nonsense_Mutation	17	7578212	7578212	G	A
**I00650**	MUC16	c.23886T>C	p.Pro7962Pro	NM_024690.2	Silent	19	9063560	9063560	A	G
**I00650**	ACVR2A	c.1310delA	p.Lys437fs	NM_001278579.1	Frame_Shift_Del	2	148683685	148683686	TA	-
**I00650**	EPHB1	c.568C>T	p.Arg190Cys	NM_004441.4	Missense_Mutation	3	134670657	134670657	C	T
**I00650**	ZIC4	c.839-1052G>A		NM_001168378.1	Intron	3	147110085	147110085	C	T
**I00650**	PBRM1	c.2128C>T	p.Arg710*	NM_018313.4	Nonsense_Mutation	3	52643768	52643768	G	A
**I00650**	FAT1	c.13727C>T	p.Thr4576Met	NM_005245.3	Missense_Mutation	4	187509786	187509786	G	A
**I00650**	SYNE1	c.8358C>A	p.His2786Gln	NM_182961.3	Missense_Mutation	6	152708336	152708336	G	T
**I00650**	PKHD1	c.9278G>A	p.Gly3093Asp	NM_138694.3	Missense_Mutation	6	51613136	51613136	C	T
**I00650**	PKHD1	c.1311G>A	p.Trp437*	NM_138694.3	Nonsense_Mutation	6	51923322	51923322	C	T
**I00650**	RIMS2	c.741C>T	p.Tyr247Tyr	NM_001100117.2	Silent	8	104897568	104897568	C	T
**I00651**	PTEN	c.1030C>T	p.Gln344*	NM_001304717.2	Nonsense_Mutation	10	89711893	89711893	C	T
**I00651**	MUC16	c.3517A>C	p.Thr1173Pro	NM_024690.2	Missense_Mutation	19	9088298	9088298	T	G
**I00651**	RHOA	c.385C>T	p.Arg129Trp	NM_001313941.1	Missense_Mutation	3	49399952	49399952	G	A
**I00651**	RHOA	c.170T>C	p.Leu57Ser	NM_001313941.1	Missense_Mutation	3	49405968	49405968	A	G
**I00651**	SYNE1	c.24313-104G>A		NM_182961.3	Intron	6	152472929	152472929	C	T
**I00651**	KMT2C	c.1012+95T>A		NM_170606.2	Intron	7	151970695	151970695	A	T
**I00651**	RIMS2	c.-208G>T		NM_001282881.1	5'UTR	8	104831528	104831528	G	T
**I00652**	TP53	c.578A>G	p.His193Arg	NM_000546.5	Missense_Mutation	17	7578271	7578271	T	C
**I00652**	MUC16	c.38202+64G>T		NM_024690.2	Intron	19	9015557	9015557	C	A
**I00652**	MUC16	c.36795+100T>G		NM_024690.2	Intron	19	9026091	9026091	A	C
**I00652**	MUC16	c.8708A>G	p.Lys2903Arg	NM_024690.2	Missense_Mutation	19	9083107	9083107	T	C
**I00652**	KMT2C	c.1012+76G>T		NM_170606.2	Intron	7	151970714	151970714	C	A
**I00653**	PTEN	c.956delT	p.Leu319fs	NM_001304717.2	Frame_Shift_Del	10	89692948	89692949	AT	-
**I00653**	ERBB2	c.689C>T	p.Pro230Leu	NM_004448.3	Missense_Mutation	17	37866384	37866384	C	T
**I00653**	MUC16	c.41476-90C>T		NM_024690.2	Intron	19	8994299	8994299	G	A
**I00653**	ERBB4	c.235-37C>A		NM_005235.2	Intron	2	212812378	212812378	G	T
**I00653**	EPHB1	c.125G>T	p.Trp42Leu	NM_004441.4	Missense_Mutation	3	134670214	134670214	G	T
**I00653**	PIK3CA	c.813+19A>G		NM_006218.3	Intron	3	178919347	178919347	A	G
**I00653**	APC	c.5065A>G	p.Thr1689Ala	NM_000038.5	Missense_Mutation	5	112176356	112176356	A	G
**I00653**	SYNE1	c.23658C>T	p.Ala7886Ala	NM_182961.3	Silent	6	152485430	152485430	G	A
**I00653**	SYNE1	c.9801A>C	p.Leu3267Phe	NM_182961.3	Missense_Mutation	6	152690113	152690113	T	G
**I00653**	SYNE1	c.1330C>T	p.Arg444Trp	NM_182961.3	Missense_Mutation	6	152804240	152804240	G	A
**I00653**	PKHD1	c.6332+56A>G		NM_138694.3	Intron	6	51777108	51777108	T	C
**I00653**	KMT2C	c.1469+59T>G		NM_170606.2	Intron	7	151949572	151949572	A	C
**I00653**	EGFR	c.1789G>T	p.Ala597Ser	NM_005228.3	Missense_Mutation	7	55233039	55233039	G	T
**I00653**	TRRAP	c.483T>G	p.Ile161Met	NM_001244580.1	Missense_Mutation	7	98493419	98493419	T	G
**I00653**	RIMS2	c.3682A>G	p.Thr1228Ala	NM_001100117.2	Missense_Mutation	8	105261807	105261807	A	G
**I00654**	MACF1	c.4629+5734T>C		NM_012090.5	Intron	1	39798759	39798759	T	C
**I00654**	CDH1	c.1051C>T	p.Gln351*	NM_004360.4	Nonsense_Mutation	16	68846080	68846080	C	T
**I00654**	SYNE1	c.17541+51G>A		NM_182961.3	Intron	6	152622953	152622953	C	T
**I00657**	SPEN	c.7015C>T	p.Arg2339Cys	NM_015001.2	Missense_Mutation	1	16259750	16259750	C	T
**I00657**	ARID1A	c.110delG	p.Gly37fs	NM_006015.4	Frame_Shift_Del	1	27022998	27022999	CG	-
**I00657**	ARID1A	c.3794G>T	p.Gly1265Val	NM_006015.4	Missense_Mutation	1	27099915	27099915	G	T
**I00657**	ARID1A	c.5236delG	p.Asp1746fs	NM_006015.4	Frame_Shift_Del	1	27105623	27105624	TG	-
**I00657**	ARID1A	c.5548delG	p.Asp1850fs	NM_006015.4	Frame_Shift_Del	1	27105930	27105931	TG	-
**I00657**	ATM	c.400G>T	p.Gly134Cys	NM_000051.3	Missense_Mutation	11	108106465	108106465	G	T
**I00657**	KRAS	c.35G>A	p.Gly12Asp	NM_033360.3	Missense_Mutation	12	25398284	25398284	C	T
**I00657**	CDH1	c.1321-35G>A		NM_004360.4	Intron	16	68849383	68849383	G	A
**I00657**	CDH1	c.1779delC	p.Ile594fs	NM_004360.4	Frame_Shift_Del	16	68855966	68855967	GC	-
**I00657**	MUC16	c.39045C>A	p.Leu13015Leu	NM_024690.2	Silent	19	9009681	9009681	G	T
**I00657**	MUC16	c.39036G>A	p.Pro13012Pro	NM_024690.2	Silent	19	9009690	9009690	C	T
**I00657**	ACVR2A	c.1310delA	p.Lys437fs	NM_001278579.1	Frame_Shift_Del	2	148683685	148683686	TA	-
**I00657**	ERBB4	c.695G>A	p.Arg232Gln	NM_005235.2	Missense_Mutation	2	212589847	212589847	C	T
**I00657**	EPHB1	c.1526G>A	p.Arg509His	NM_004441.4	Missense_Mutation	3	134880963	134880963	G	A
**I00657**	ZIC4	c.565G>A	p.Ala189Thr	NM_001168378.1	Missense_Mutation	3	147113912	147113912	C	T
**I00657**	MLH1	c.1558+1G>A		NM_000249.3	Intron	3	37070424	37070424	G	A

**I00657**	FAT1	c.10293C>T	p.Ser3431Ser	NM_005245.3	Silent	4	187527281	187527281	G	A
**I00657**	FAT1	c.9456C>T	p.Ala3152Ala	NM_005245.3	Silent	4	187534270	187534270	G	A
**I00657**	SYNE1	c.25286T>C	p.Leu8429Ser	NM_182961.3	Missense_Mutation	6	152461257	152461257	A	G
**I00657**	KMT2C	c.12308A>G	p.Asp4103Gly	NM_170606.2	Missense_Mutation	7	151850008	151850008	T	C
**I00657**	TRRAP	c.9035-93T>C		NM_001244580.1	Intron	7	98581623	98581623	T	C
**I00658**	TP53	c.782+1G>A		NM_000546.5	Intron	17	7577498	7577498	C	T
**I00658**	MUC16	c.32776G>T	p.Val10926Phe	NM_024690.2	Missense_Mutation	19	9048855	9048855	C	A
**I00658**	EPHB1	c.1223C>T	p.Ala408Val	NM_004441.4	Missense_Mutation	3	134851817	134851817	C	T
**I00658**	FAT4	c.10894G>A	p.Gly3632Ser	NM_001291303.1	Missense_Mutation	4	126373059	126373059	G	A
**I00658**	SYNE1	c.12226-109G>T		NM_182961.3	Intron	6	152660610	152660610	C	A
