# Supplementary material for: Liquid Biopsy in Gastric Cancer: Analysis of Somatic Cancer Tissue Mutations in Plasma Cell-Free DNA for Predicting Disease State and Patient Survival
Source: Clin Transl Gastroenterol. 2021 Sep 24;12(9):e00403. doi: 10.14309/ctg.0000000000000403 (PMC8462609; doi:10.14309/ctg.0000000000000403)
Supplement: SUPPLEMENTARY MATERIAL [file ct9-12-e00403-s007.pdf]

Supplementary table 5. Somatic variants in plasma cfDNA of GC patients

Sample name	Gene	cHGVS	pHGVS	Transcript	Variant classification	Chr	Start	End	REF	ALT
**I00629**	KMT2C	c.11670+68T>C		NM_170606.2	Intron	7	151855880	151855880	A	G
**I00629**	KMT2C	c.2961C>G	p.Tyr987*	NM_170606.2	Nonsense_Mutation	7	151927023	151927023	G	C
**I00629**	KMT2C	c.2917A>G	p.Arg973Gly	NM_170606.2	Missense_Mutation	7	151927067	151927067	T	C
**I00629**	KMT2C	c.2652+62A>G		NM_170606.2	Intron	7	151935730	151935730	T	C
**I00629**	KMT2C	c.1017G>C	p.Lys339Asn	NM_170606.2	Missense_Mutation	7	151962290	151962290	C	G
**I00629**	SYNE1	c.10145+99C>T		NM_182961.3	Intron	6	152685883	152685883	G	A
**I00630**	KMT2C	c.5009-19C>T		NM_170606.2	Intron	7	151882735	151882735	G	A
**I00630**	KMT2C	c.5009-59T>A		NM_170606.2	Intron	7	151882775	151882775	A	T
**I00630**	KMT2C	c.2652+62A>G		NM_170606.2	Intron	7	151935730	151935730	T	C
**I00630**	KMT2C	c.1012+76G>T		NM_170606.2	Intron	7	151970714	151970714	C	A
**I00630**	MUC16	c.42395+25C>T		NM_024690.2	Intron	19	8976556	8976556	G	A
**I00630**	MUC16	c.40664C>T	p.Pro13555Leu	NM_024690.2	Missense_Mutation	19	8999511	8999511	G	A
**I00631**	KMT2C	c.5009-19C>T		NM_170606.2	Intron	7	151882735	151882735	G	A
**I00631**	KMT2C	c.2961C>G	p.Tyr987*	NM_170606.2	Nonsense_Mutation	7	151927023	151927023	G	C
**I00631**	KMT2C	c.2917A>G	p.Arg973Gly	NM_170606.2	Missense_Mutation	7	151927067	151927067	T	C
**I00631**	KMT2C	c.2652+62A>G		NM_170606.2	Intron	7	151935730	151935730	T	C
**I00631**	MUC16	c.36568+19G>A		NM_024690.2	Intron	19	9028205	9028205	C	T
**I00631**	MUC16	c.36568+10T>C		NM_024690.2	Intron	19	9028214	9028214	A	G
**I00632**	KMT2C	c.5009-19C>T		NM_170606.2	Intron	7	151882735	151882735	G	A
**I00632**	KMT2C	c.5009-59T>A		NM_170606.2	Intron	7	151882775	151882775	A	T
**I00632**	KMT2C	c.2652+62A>G		NM_170606.2	Intron	7	151935730	151935730	T	C
**I00632**	KMT2C	c.1012+13C>T		NM_170606.2	Intron	7	151970777	151970777	G	A
**I00632**	KMT2C	c.850-17T>G		NM_170606.2	Intron	7	151970969	151970969	A	C
**I00633**	KMT2C	c.5009-19C>T		NM_170606.2	Intron	7	151882735	151882735	G	A
**I00633**	KMT2C	c.5009-59T>A		NM_170606.2	Intron	7	151882775	151882775	A	T
**I00633**	KMT2C	c.2652+62A>G		NM_170606.2	Intron	7	151935730	151935730	T	C
**I00634**	KMT2C	c.5009-19C>T		NM_170606.2	Intron	7	151882735	151882735	G	A
**I00634**	KMT2C	c.5009-59T>A		NM_170606.2	Intron	7	151882775	151882775	A	T
**I00634**	KMT2C	c.2652+62A>G		NM_170606.2	Intron	7	151935730	151935730	T	C
**I00634**	MUC16	c.39206+28T>C		NM_024690.2	Intron	19	9009239	9009239	A	G
**I00634**	MUC16	c.39206+23A>G		NM_024690.2	Intron	19	9009244	9009244	T	C
**I00636**	KMT2C	c.5068A>C	p.Arg1690Arg	NM_170606.2	Silent	7	151882657	151882657	T	G
**I00636**	KMT2C	c.2968A>G	p.Ser990Gly	NM_170606.2	Missense_Mutation	7	151927016	151927016	T	C
**I00636**	KMT2C	c.1005T>A	p.Pro335Pro	NM_170606.2	Silent	7	151970797	151970797	A	T
**I00636**	KMT2C	c.925C>T	p.Pro309Ser	NM_170606.2	Missense_Mutation	7	151970877	151970877	G	A
**I00636**	MUC16	c.40664C>T	p.Pro13555Leu	NM_024690.2	Missense_Mutation	19	8999511	8999511	G	A
**I00636**	MUC16	c.40644T>C	p.Asp13548Asp	NM_024690.2	Silent	19	8999531	8999531	A	G
**I00638**	KMT2C	c.5009-19C>T		NM_170606.2	Intron	7	151882735	151882735	G	A
**I00638**	KMT2C	c.2652+62A>G		NM_170606.2	Intron	7	151935730	151935730	T	C
**I00638**	KMT2C	c.2418C>G	p.Ser806Ser	NM_170606.2	Silent	7	151945101	151945101	G	C
**I00639**	KMT2C	c.5009-19C>T		NM_170606.2	Intron	7	151882735	151882735	G	A
**I00639**	KMT2C	c.5009-59T>A		NM_170606.2	Intron	7	151882775	151882775	A	T
**I00639**	KMT2C	c.2652+62A>G		NM_170606.2	Intron	7	151935730	151935730	T	C
**I00642**	MACF1	c.14695G>T	p.Ala4899Ser	NM_012090.5	Missense_Mutation	1	39919508	39919508	G	T
**I00643**	KMT2C	c.5009-19C>T		NM_170606.2	Intron	7	151882735	151882735	G	A
**I00643**	KMT2C	c.5009-59T>A		NM_170606.2	Intron	7	151882775	151882775	A	T
**I00643**	KMT2C	c.2652+62A>G		NM_170606.2	Intron	7	151935730	151935730	T	C
**I00643**	MUC16	c.40611-16A>C		NM_024690.2	Intron	19	8999580	8999580	T	G
**I00645**	KMT2C	c.5009-19C>T		NM_170606.2	Intron	7	151882735	151882735	G	A
**I00645**	KMT2C	c.5009-59T>A		NM_170606.2	Intron	7	151882775	151882775	A	T
**I00645**	KMT2C	c.1012+76G>T		NM_170606.2	Intron	7	151970714	151970714	C	A
**I00645**	MUC16	c.40734C>T	p.Ile13578Ile	NM_024690.2	Silent	19	8999441	8999441	G	A
**I00645**	MUC16	c.40732A>G	p.Ile13578Val	NM_024690.2	Missense_Mutation	19	8999443	8999443	T	C
**I00645**	MUC16	c.40713G>A	p.Leu13571Leu	NM_024690.2	Silent	19	8999462	8999462	C	T
**I00645**	MUC16	c.40707G>C	p.Trp13569Cys	NM_024690.2	Missense_Mutation	19	8999468	8999468	C	G
**I00645**	TP53	c.467delG	p.Arg156fs	NM_000546.5	Frame_Shift_Del	17	7578462	7578463	GC	-
**I00647**	KMT2C	c.5009-59T>A		NM_170606.2	Intron	7	151882775	151882775	A	T
**I00647**	KMT2C	c.2961C>G	p.Tyr987*	NM_170606.2	Intron	7	151921037	151921037	G	T
**I00647**	KMT2C	c.2917A>G	p.Arg973Gly	NM_170606.2	Nonsense_Mutation	7	151927023	151927023	G	C
**I00647**	KMT2C	c.2652+62A>G		NM_170606.2	Missense_Mutation	7	151927067	151927067	T	C
**I00647**	KMT2C	c.1012+103T>G		NM_170606.2	Intron	7	151935730	151935730	T	C

**I00647**	KMT2C	c.3323+63C>A		NM_170606.2	Intron	7	151970687	151970687	A	C
**I00647**	MUC16	c.38577C>A	p.Leu12859Leu	NM_024690.2	Silent	19	9012867	9012867	G	T
**I00648**	KMT2C	c.5009-19C>T		NM_170606.2	Intron	7	151882735	151882735	G	A
**I00648**	KMT2C	c.5009-59T>A		NM_170606.2	Intron	7	151882775	151882775	A	T
**I00648**	KMT2C	c.2769+5G>T		NM_170606.2	Intron	7	151932897	151932897	C	A
**I00648**	KMT2C	c.2652+62A>G		NM_170606.2	Intron	7	151935730	151935730	T	C
**I00648**	MUC16	c.37195C>T	p.Leu12399Leu	NM_024690.2	Silent	19	9021128	9021128	G	A
**I00649**	KMT2C	c.5009-19C>T		NM_170606.2	Intron	7	151882735	151882735	G	A
**I00649**	KMT2C	c.5009-59T>A		NM_170606.2	Intron	7	151882775	151882775	A	T
**I00649**	KMT2C	c.2769+83A>T		NM_170606.2	Intron	7	151926977	151926977	A	G
**I00649**	KMT2C	c.2652+62A>G		NM_170606.2	Intron	7	151932819	151932819	T	A
**I00649**	KMT2C	c.1184+61T>C		NM_170606.2	Intron	7	151935730	151935730	T	C
**I00649**	KMT2C	c.1012+80G>T		NM_170606.2	Intron	7	151962062	151962062	A	G
**I00649**	KMT2C	c.850-63C>T		NM_170606.2	Intron	7	151970710	151970710	C	A
**I00649**	KMT2C	c.2976+31T>C		NM_170606.2	Intron	7	151971015	151971015	G	A
**I00649**	MUC16	c.38948-7T>C		NM_024690.2	Intron	19	9010720	9010720	A	G
**I00650**	KMT2C	c.2652+62A>G		NM_170606.2	Intron	7	151935730	151935730	T	C
**I00651**	KMT2C	c.5009-19C>T		NM_170606.2	Intron	7	151882735	151882735	G	A
**I00651**	KMT2C	c.5009-59T>A		NM_170606.2	Intron	7	151882775	151882775	A	T
**I00651**	KMT2C	c.1012+95T>A		NM_170606.2	Intron	7	151970695	151970695	A	T
**I00652**	KMT2C	c.5009-19C>T		NM_170606.2	Intron	7	151882735	151882735	G	A
**I00652**	KMT2C	c.1012+76G>T		NM_170606.2	Intron	7	151970714	151970714	C	A
**I00653**	KMT2C	c.5009-19C>T		NM_170606.2	Intron	7	151882735	151882735	G	A
**I00653**	KMT2C	c.2961C>G	p.Tyr987*	NM_170606.2	Nonsense_Mutation	7	151927023	151927023	G	C
**I00653**	KMT2C	c.2917A>G	p.Arg973Gly	NM_170606.2	Missense_Mutation	7	151927067	151927067	T	C
**I00653**	KMT2C	c.2755G>C	p.Val919Leu	NM_170606.2	Missense_Mutation	7	151932916	151932916	C	G
**I00653**	KMT2C	c.2652+62A>G		NM_170606.2	Intron	7	151935730	151935730	T	C
**I00657**	ACVR2A	c.1310delA	p.Lys437fs	NM_001278579.1	Frame_Shift_Del	2	148683685	148683686	TA	-
**I00657**	ERBB4	c.695G>A	p.Arg232Gln	NM_005235.2	Missense_Mutation	2	212589847	212589847	C	T
**I00657**	FAT1	c.10293C>T	p.Ser3431Ser	NM_005245.3	Silent	4	187527281	187527281	G	A
**I00657**	KMT2C	c.5009-19C>T		NM_170606.2	Missense_Mutation	7	151850008	151850008	T	C
**I00657**	KMT2C	c.5009-59T>A		NM_170606.2	Intron	7	151882735	151882735	G	A
**I00657**	KMT2C	c.2769+83A>T		NM_170606.2	Intron	7	151882775	151882775	A	T
**I00657**	KMT2C	c.2652+62A>G		NM_170606.2	Intron	7	151932819	151932819	T	A
**I00657**	KMT2C	c.12308A>G	p.Asp4103Gly	NM_170606.2	Intron	7	151935730	151935730	T	C
**I00657**	MLH1	c.1558+1G>A		NM_000249.3	Intron	3	37070424	37070424	G	A
**I00657**	MUC16	c.39045C>A	p.Leu13015Leu	NM_024690.2	Silent	19	9009681	9009681	G	T
**I00657**	MUC16	c.39036G>A	p.Pro13012Pro	NM_024690.2	Silent	19	9009690	9009690	C	T
**I00657**	PREX2	c.4605-27C>T		NM_024870.3	Intron	8	69129824	69129824	C	T
**I00657**	SPEN	c.7015C>T	p.Arg2339Cys	NM_015001.2	Missense_Mutation	1	16259750	16259750	C	T
**I00657**	SYNE1	c.25286T>C	p.Leu8429Ser	NM_182961.3	Missense_Mutation	6	152461257	152461257	A	G
**I00658**	FAT4	c.10894G>A	p.Gly3632Ser	NM_001291303.1	Missense_Mutation	4	126373059	126373059	G	A
**I00658**	KMT2C	c.2652+62A>G		NM_170606.2	Intron	7	151935730	151935730	T	C
**I00658**	KMT2C	c.2533-102G>T		NM_170606.2	Intron	7	151936013	151936013	C	A
**I00658**	KMT2C	c.1017G>C	p.Lys339Asn	NM_170606.2	Missense_Mutation	7	151962290	151962290	C	G
**I00658**	MUC16	c.39206+39C>A		NM_024690.2	Intron	19	9009228	9009228	G	T
**I00658**	MUC16	c.37547-8T>C		NM_024690.2	Intron	19	9009349	9009349	C	G
**I00658**	MUC16	c.37547-13_37547-12delCAinsAG		NM_024690.2	Intron	19	9019348	9019348	A	G
**I00658**	MUC16	c.39139-15G>C		NM_024690.2	Intron	19	9019352	9019352	TG	CT
**I00658**	PKHD1	c.729A>G	p.Ala243Ala	NM_138694.3	Silent	6	51934304	51934304	T	C
**I00658**	SYNE1	c.10145+42A>G		NM_182961.3	Intron	6	152660610	152660610	C	A
**I00658**	SYNE1	c.12226-109G>T		NM_182961.3	Intron	6	152685940	152685940	T	C
**I00658**	TP53	c.782+1G>A		NM_000546.5	Intron	17	7577498	7577498	C	T

[1] Variants in *KMT2C* that were recurrently called in many samples could be potential false-positives despite UMI-based error correction, e.g. c.2652+62A>G, c.5009-59T>A, c.5009-19C>T.
