# Supplementary material for: Liquid Biopsy in Gastric Cancer: Analysis of Somatic Cancer Tissue Mutations in Plasma Cell-Free DNA for Predicting Disease State and Patient Survival
Source: Clin Transl Gastroenterol. 2021 Sep 24;12(9):e00403. doi: 10.14309/ctg.0000000000000403 (PMC8462609; doi:10.14309/ctg.0000000000000403)
Supplement: SUPPLEMENTARY MATERIAL [file ct9-12-e00403-s008.pdf]

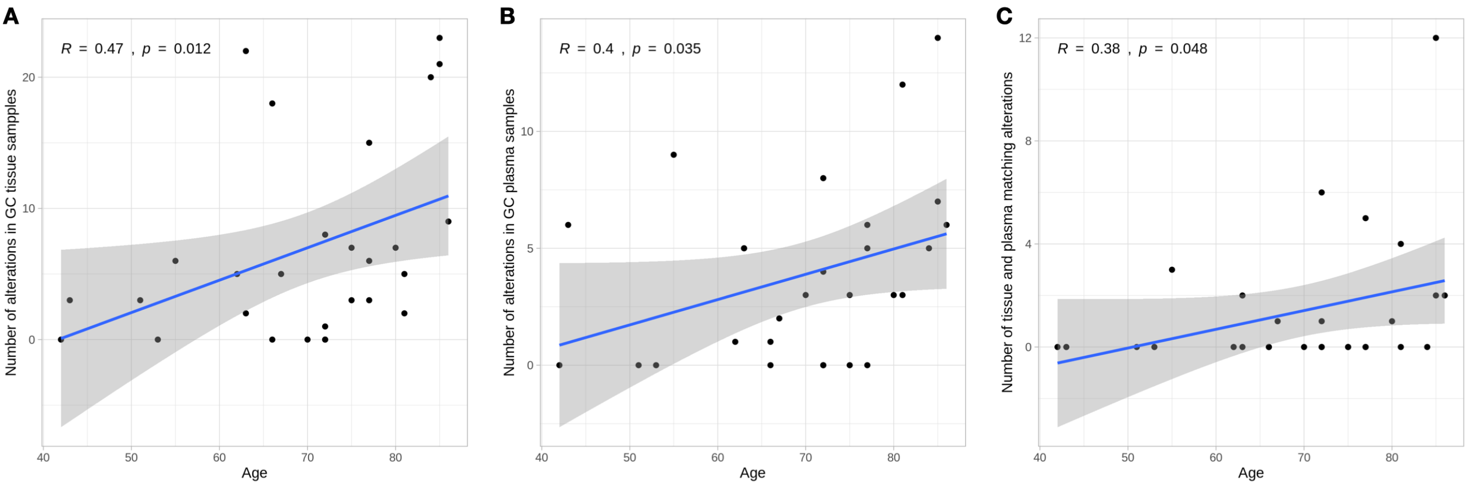

Supplementary Figure 4. Spearman's correlation analysis between the quantity of alterations with patients' age **a** in tissue samples; **b** plasma samples; and **c** tissue and plasma matching variants. The quantity of unique somatic alterations detected in tissue, plasma and the quantity of tissue matching alterations in plasma revealed positive moderate correlation with age (R = 0.47, p = 0.012; R = 0.4, p-value = 0.035; R = 0.38, p = 0.048, respectively)
