# Supplementary material for: Liquid Biopsy in Gastric Cancer: Analysis of Somatic Cancer Tissue Mutations in Plasma Cell-Free DNA for Predicting Disease State and Patient Survival
Source: Clin Transl Gastroenterol. 2021 Sep 24;12(9):e00403. doi: 10.14309/ctg.0000000000000403 (PMC8462609; doi:10.14309/ctg.0000000000000403)
Supplement: SUPPLEMENTARY MATERIAL [file ct9-12-e00403-s009.pdf]

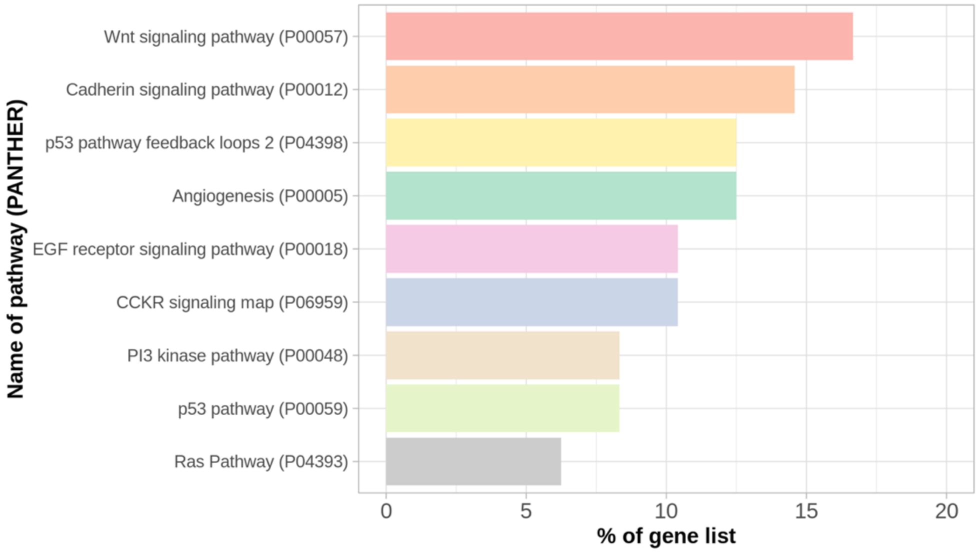


**Supplementary Figure 3.** Gene list pathway enrichment analysis according to PANTHER (Protein Analysis Through Evolutionary Relationships). 26 genes of our 38-gene were assigned to nine different pathways annotated in PANTHER classification system; 12 genes were unclassified. Mutated genes were mostly associated with Wnt and Cadherin signalling pathways
